# Ionic Liquid Meets MOF: A Facile Method to Optimize the Structure of CoSe2‐NiSe2 Heterojunctions with N, P, and F Triple‐Doped Carbon Using Ionic Liquid for Efficient Hydrogen Evolution and Flexible Supercapacitors

**DOI:** 10.1002/advs.202206029

**Published:** 2023-01-13

**Authors:** Mingjie Yi, Jiayu Ma, Yi Ren, Hao Wang, Lin Xie, Zhenye Zhu, Jiaheng Zhang

**Affiliations:** ^1^ State Key Laboratory of Advanced Welding and Joining Harbin Institute of Technology Shenzhen 518055 P. R. China; ^2^ Research Centre of Printed Flexible Electronics School of Materials Science and Engineering Harbin Institute of Technology Shenzhen 518055 P. R. China

**Keywords:** flexible supercapacitors, hydrogen evolution, ionic liquid, metal‐organic frameworks

## Abstract

The rational design of catalysts’ spatial structure is vitally important to boost catalytic performance by exposing the active sites and increasing specific surface area. Herein, the heteroatom doping and morphology of CoNi metal‐organic frameworks(MOF) are modulated by controlling the volume of ionic liquid used in synthesis and generating CoSe_2_‐NiSe_2_ heterojunction structures wrapped by N, P, F tri‐doped carbon(NPFC) after a selenisation process. Notably, the unique cubic porous structure of CoSe_2_‐NiSe_2_/NPFC results in a specific surface five times that of the sheet‐like hollow structure produced without ionic liquid. Moreover, the charge redistribution during heterojunction formation is verified in detail using synchrotron radiation. Density functional theory calculations reveal that the formation of heterojunctions and doping of heteroatoms successfully lower the *ΔG*_H*_ and *ΔG*_OH*_ values. Consequently, CoSe_2_‐NiSe_2_/NPFC exhibits excellent activity for HER in both acidic and alkaline solutions. Meanwhile, CoSe_2_‐NiSe_2_/NPFC as a cathode material exhibits excellent performance in a flexible solid‐state supercapacitor, with a superior energy density of 55.7 Wh kg^−1^ at an extremely high‐power density of 15.9 kW kg^−1^. This material design provides new ideas for not only using ionic liquids to modulate the morphology of MOFs but also deriving heterojunctions and heteroatom‐doped carbon from MOFs.

## Graphical abstract

A series of Co‐Ni‐based MOFs with different morphologies qre prepared by varying the volume of injected ionic liquid (IL). Among them, CoSe_2_‐NiSe_2_/NPFC exhibits larger specific surface area nearly five times and more heteroatoms doping than that of CoSe_2_‐NiSe_2_/C (synthesised without adding IL). After testing, CoSe_2_‐NiSe_2_/NPFC has excellent performance in hydrogen evolution and supercapacitor.

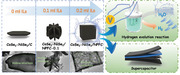

## 1 Introduction

To alleviate the growing energy crisis and environmental pollution, numerous researchers have focused on advanced energy storage technologies and sustainable energy sources.^[^ ^1^, ^2^, ^3^, ^4^, ^5^, ^6^ ^]^ Hydrogen as a sustainable energy storage medium with the highest gravimetric energy density has garnered significant attention for potentially replacing fossil fuels.^[^ ^7^, ^8^, ^9^ ^]^ High‐purity hydrogen can be produced easily using water electrolysis, but the best Pt‐based electrocatalysts used in this process suffer from high costs and the scarcity of platinum. Supercapacitors are another promising option for energy storage because of their low cost, quick charge‐discharge process, and long cycling stability.^[^ ^10^, ^11^, ^12^, ^13^, ^14^, ^15^ ^]^ Nevertheless, the energy density of supercapacitors is still insufficient.

Transition metal selenides (TMSs) may overcome this bottleneck owing to their advantages over the corresponding transition metal oxides/hydrides and sulfides, namely a low energy band, good metal‐like properties, significant conductivity, and good electrochemical properties.^[^ ^16^ ^]^ Additionally, TMS does not create polyselenide intermediates during the charge/discharge cycle, and this has been cited as evidence for their higher cycling stability. Thus, TMSs are regarded as promising electrode materials and catalysts owing to their higher electrochemical performance. TMSs, such as MoSe_2_,^[^ ^17^ ^]^ NiSe_2_,^[^ ^18^ ^]^ and CoSe_2_,^[^ ^19^ ^]^ exhibit moderately high electrochemical properties when used as battery‐type electrode materials. Nevertheless, preparing TMS materials with a large specific capacity, exceptional rate characteristics, and admirable cycling stability is challenging. Previous studies have reported that the heterojunctions of bimetallic selenides typically have better electrochemical characteristics than monometallic selenides owing to the synergy among various chemically bonded metal ions.^[^ ^20^, ^21^, ^22^, ^23^, ^24^ ^]^ First, charge transfer at the heterojunction interface causes electron redistribution, which affects the electronic structure of the components. Second, heterojunctions can expose more edges in the nanostructure to provide abundant reaction sites.^[^ ^25^, ^26^, ^27^, ^28^, ^29^, ^30^, ^31^, ^32^ ^]^ For example, Zhu et al. prepared a bimetallic selenide NiSe_2_/CoSe_2_ via a simple hydrothermal process, and the selenide exhibited a larger specific capacity (171.5 mAh g^−1^ at 1.0 A g^−1^) compared with pristine CoSe_2_ and NiSe_2_.^[^ ^33^ ^]^ Zhong et al. reported a crystalline‐amorphous CoSe_2_/CoP heterojunction that required an overpotential of 65 mV to drive a current density of 10 mA cm^−2^ in an acidic solution.^[^ ^16^ ^]^ Owing to synergistic effects, bimetallic selenides perform better than the corresponding monometallic selenides in HER and supercapacitors. A reasonable construction of bimetallic selenides with advanced structures is among the best strategies to improve the electrochemical properties of electrode materials, yet the optimization of these structures is challenging.

Owing to their distinctive architecture and properties, metal‐organic frameworks (MOFs) have attracted growing interest and applications in the energy and environmental domains.^[^ ^34^, ^35^, ^36^, ^37^ ^]^ Particularly, numerous MOFs are suitable precursors for the synthesis of heterogeneous structures.^[^ ^38^, ^39^, ^40^ ^]^ Ionic liquids (ILs), which consist of anions and cations and exhibit a liquid state at near room temperature, have desirable properties, such as non‐flammability, nontoxicity, and easy recovery. During material synthesis, ILs frequently provide heteroatom dopants and/or act as structurally oriented templates. Surface alteration and IL adsorption can inhibit long‐range crystallographic order, which in turn may reduce the free energy and further constrain the growth of a particular crystallographic plane. For instance, doping with 1‐butyl‐3‐methylimidazolium hexafluorophosphate ([hmim][PF_6_]) created an amorphous structure and larger microspheres consisting of thicker CoFe_2_O_4_ sheets.^[^ ^41^ ^]^ However, there are very few studies to modulate the morphology of MOF by ionic liquids.

This work aimed to improve the electrochemical properties of CoSe_2_‐NiSe_2_ synthesized from a CoNi‐based MOF precursor. The heteroatom doping concentrations and morphology of the MOF could be adjusted by using different volumes of [hmim][PF_6_] in synthesis. After a selenization process, the CoSe_2_‐NiSe_2_ was wrapped by N, P, and F tri‐doped carbon to form a heterojunction structure (CoSe_2_‐NiSe_2_/NPFC) that offers numerous advantages. First, its porous structure increased the specific surface area by nearly 400% compared with that synthesized without added IL. Second, the introduction of P, N, and F dopants from the IL resulted in more exposed active sites in the carbon material and also enhanced the electronegativity, thereby improving the ability of CoSe_2_‐NiSe_2_/NPFC to collect protons. Third, charge transfer at the CoSe_2_‐NiSe_2_ heterojunction interface affected electron distribution and the electronic structure of each component; consequently, the performances in HER and supercapacitors were improved. In addition, we used synchrotron radiation to compare the Ni K‐edge and Co K‐edge in CoSe_2_‐NiSe_2_/NPFC, CoSe_2_/NPFC, and NiSe_2_/NPFC to reveal the charge transfer pathway. Density functional theory (DFT) calculation verified that the heterojunction and N, P, F tri‐doped carbon in CoSe_2_‐NiSe_2_/NPFC can modulate the electronic structure, and the resultant lower energy barriers of OH^*^/H^*^ adsorption improve the HER and supercapacitor performance. Thus, this study further explores the use of ILs to modulate the morphology of MOF and provides new ideas for deriving heterojunctions and heteroatom‐doped carbon from MOFs.

## 2 Results and Discussion

### 2.1 Structure and Component Analysis

**Figure 1:**
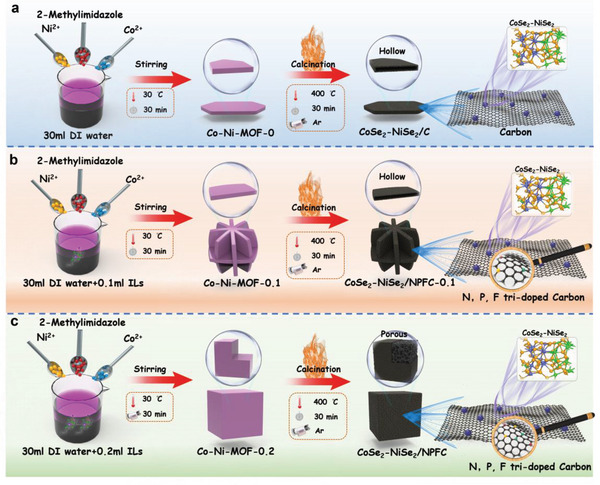
Schematic of preparation processes of a) CoSe_2_‐NiSe_2_/C, b) CoSe_2_‐NiSe_2_/NPFC‐0.1, and c) CoSe_2_‐NiSe_2_/NPFC.

**Figure** **1** illustrates the method for modulating the morphology of CoSe_2_‐NiSe_2_ and carbon complexes by varying the volume of IL (0, 0.1, and 0.2 ml). CoSe_2_‐NiSe_2_/C prepared without added IL exhibited a hollow sheet‐like structure (Figure 1a). After introducing 0.1 ml IL, CoSe_2_‐NiSe_2_/NPFC‐0.1 exhibited a sphere‐like structure with aggregated hollow lamellae (Figure 1b). When using 0.2 ml IL, the synthesized CoSe_2_‐NiSe_2_/NPFC‐0.2 (hereinafter referred to as “CoSe_2_‐NiSe_2_/NPFC”) featured a cubic particle shape with a porous interior (Figure 1c).

**Figure 2:**
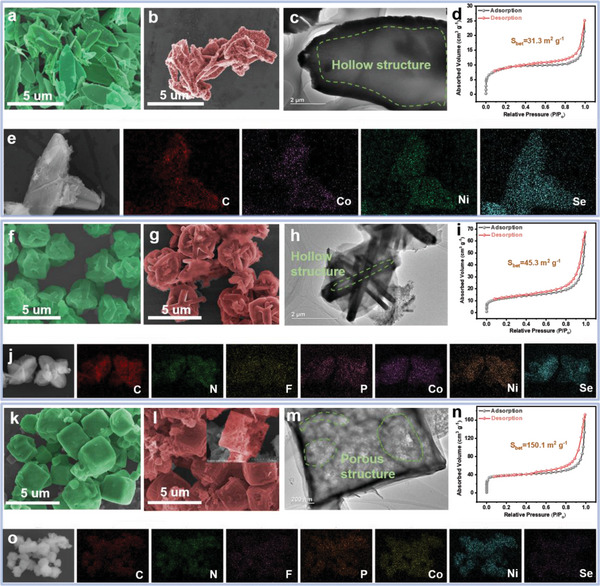
Scanning electron microscopy (SEM) images of a) Co‐Ni‐MOF‐0 (in green false color) and b) CoSe_2_‐NiSe_2_/C (in red false color). c) Transmission electron microscopy (TEM) image, d) Brunauer–Emmett–Teller (BET) data, and e) elemental mapping results of CoSe_2_‐NiSe_2_/C. SEM images of f) Co‐Ni‐MOF‐0.1 and g) CoSe_2_‐NiSe_2_/NPFC‐0.1. h) TEM image, i) BET data, and j) elemental mapping results of CoSe_2_‐NiSe_2_/NPFC‐0.1. SEM images of k) Co‐Ni‐MOF‐0.2 and l) CoSe_2_‐NiSe_2_/NPFC. m) TEM image, n) BET data, and o) elemental mapping results of CoSe_2_‐NiSe_2_/NPFC.

Figure 2a,b show the scanning electron microscopy (SEM) images of Co‐Ni‐MOF‐0 and CoSe_2_‐NiSe_2_/C with a sheet‐like structure, respectively. **Figure** **2**c shows the interior hollow structure of CoSe_2_‐NiSe_2_/C, which explains its large specific surface area of 31.3 m^2^ g^−1^ according to the Brunauer–Emmett–Teller (BET) measurement (Figure 2d). The distribution of C, Co, Ni, and Se elements was revealed by energy dispersive spectroscopy (EDS) analysis and elemental mapping (Figure 2e and Figure S1a, Supporting Information). Figure 2f,g displays the SEM images of Co‐Ni‐MOF‐0.1 and CoSe_2_‐NiSe_2_/NPFC‐0.1, respectively. The addition of 0.1 ml IL during synthesis changed the morphology of Co‐Ni‐MOF into a sphere‐like structure with self‐assembled lamellae. The transmission electron microscopy (TEM) image also revealed a hollow structure (Figure 2h), and the specific surface area was 45.3 m^2^ g^−1^ (Figure 2i). EDS and elemental mapping results confirmed the successful doping of N, P, and F elements in CoSe_2_‐NiSe_2_/NPFC‐0.1 from the IL (Figure 2j and Figure S1b, Supporting Information). Upon increasing the volume of added IL to 0.2 ml, the morphology of Co‐Ni‐MOF‐0.2 further changed to a cubic shape (Figure 2k). After selenization, CoSe_2_‐NiSe_2_/NPFC maintained its cubic morphology, and its interior was porous (Figure 2l). This porous structure was also confirmed by TEM observation (Figure 2m). Compared with CoSe_2_‐NiSe_2_/NPFC‐0.1 and CoSe_2_‐NiSe_2_/C, the BET‐specific surface area of CoSe_2_‐NiSe_2_/NPFC was significantly larger, which suggests that it may contain more abundant active sites for electrochemical reactions (Figure 2n). According to the EDS data and mapping, the N, P, and F elements were also successfully doped into CoSe_2_‐NiSe_2_/NPFC, and their concentrations are higher than those in CoSe_2_‐NiSe_2_/NPFC‐0.1 (Figure S1c, Supporting Information and Figure 2o). The above results demonstrate that the morphology of MOF could be adjusted through the volume of IL injected during synthesis. The likely reason is that ILs can significantly inhibit the long‐range crystalline order and alter material growth. Additionally, we found that CoSe_2_‐NiSe_2_/NPFC has lower water contact angles than CoSe_2_‐NiSe_2_/C, thus indicating a more hydrophilic surface that facilitates reactions with the electrolyte (Figure S2, Supporting Information).

To better compare the effects of heterojunctions on the electrochemical properties, CoSe_2_/NPFC and NiSe_2_/NPFC were prepared using similar methods, and their elemental composition was verified by EDS (Figure S3a,b, Supporting Information). However, these two monometallic composites failed to self‐assemble into a cubic structure (Figure S4a,b, Supporting Information).

**Figure 3:**
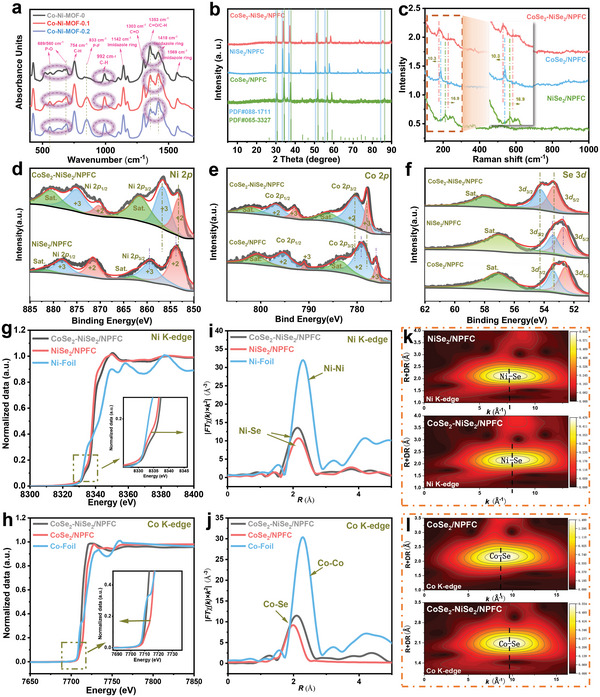
a) FT‐IR spectra of Co‐Ni‐MOF‐0, Co‐Ni‐MOF‐0.1, and Co‐Ni‐MOF‐0.2. b) X‐ray diffraction (XRD) patterns and c) Raman spectra of CoSe_2_‐NiSe_2_/NPFC, CoSe_2_/NPFC, and NiSe_2_/NPFC. XPS peaks of d) Ni 2*p*, e) Co 2*p*, and f) Se 3*d* for CoSe_2_‐NiSe_2_/NPFC, NiSe_2_/NPFC, and Ni foil. XANES spectra at the g) Ni K‐edge and h) Co K‐edge for CoSe_2_‐NiSe_2_/NPFC and other materials. The corresponding FT EXAFS data at the i) Ni K‐edge and j) Co K‐edge for CoSe_2_‐NiSe_2_/NPFC and other materials. WT EXAFS plots at the k) Ni K‐edge and l) Co K‐edge for CoSe_2_‐NiSe_2_/NPFC and other materials.

Although modulation of material morphology by ILs has been reported before, the underlying mechanism has rarely been studied. Herein, Fourier‐transform infrared (FT‐IR) spectroscopy was used to thoroughly examine changes in chemical bonding after adding different amounts of IL (**Figure** **3**a). The characteristic peaks of CoSe_2_‐NiSe_2_/NPFC‐0.1 and CoSe_2_‐NiSe_2_/NPFC were similar to that of CoSe_2_‐NiSe_2_/C, whereas the relative intensities of some peaks changed significantly. In particular, the imidazole ring vibration at 1418 cm^−1^ increased substantially with the introduction of [hmim][PF_6_], and the C=O stretching peak at 1353 cm^−1^ became weaker owing to deprotonation coordination of the carboxyl group. The absorption peak at 833 cm^−1^ is assigned to P–F vibration and its intensity increased significantly to indicate the successful introduction of P–F bonds. The origin of the absorption peaks at 560 and 689 cm^−1^ is difficult to determine, but they may be closely related to the structure of P–O, thus indicating the possible existence of phosphate and hydroxyl/carboxylate coordination in the IL. Overall, N, P, and F atoms were successfully introduced into the synthesized material. The introduction of N was mainly associated with the imidazole ring coordinating with the carboxyl group on the backbone of the precursor molecule. In contrast, the introduction of P and F may be closely related to the ligand exchange reaction between phosphate and hydroxyl/carboxyl groups of the precursor. Therefore, we assumed that these heteroatoms from the IL became coordinated with the MOF and changed the latter's morphology in a dose‐dependent manner.

The occurrence of heterojunctions between CoSe_2_ and NiSe_2_ was analyzed using a number of experimental methods. High‐resolution TEM was used to investigate the presence of heterojunctions in CoSe_2_‐NiSe_2_/NPFC (Figure S5, Supporting Information). X‐ray diffraction (XRD) analysis of CoSe_2_‐NiSe_2_/NPFC revealed that it contained both NiSe_2_ (088‐1711) and CoSe_2_ (065‐3227), as shown in Figure 3b. Additionally, all diffraction peaks of CoSe_2_‐NiSe_2_/NPFC coincided with those of CoSe_2_ and NiSe_2_. These results confirmed the presence of NiSe_2_ and CoSe_2_ heterojunctions in CoSe_2_‐NiSe_2_/NPFC. In the Raman spectrum of CoSe_2_‐NiSe_2_/NPFC, the characteristic peak at 189.2 cm^−1^ agrees well with that of CoSe_2_ (Figure 3c), whereas that at 209.1 cm^−1^ is attributed to the *T*_g_ peak of NiSe_2_. Most significantly, the characteristic peak of NiSe_2_ exhibits a minor blue shift from 209.1 to 217.0 cm^−1^ compared with CoSe_2_‐NiSe_2_/NPFC, whereas the peak of CoSe_2_ exhibits a slight red shift. The opposite shifts, which indicate a diamagnetic charge transfer between NiSe_2_ and CoSe_2_, were produced via the heterojunctions formed between NiSe_2_ and CoSe_2_.

X‐ray photoelectron spectroscopy (XPS) was used to examine the chemical states of the elements and the interactions between them. The high‐resolution Ni 2*p* and Co 2*p* spectra exhibited four primary peaks and two satellite peaks of Ni or Co (Figure 3d,e, respectively). For CoSe_2_‐NiSe_2_/NPFC, Ni^2+^ and Ni^3+^ of Ni 2*p*_3/2_ were responsible for the peaks at 853.3 and 856.7 eV, whereas Ni^2+^ and Ni^3+^ of Ni 2*p*_1/2_ were responsible for the peaks at 870.30 and 875.2 eV, respectively. Similarly, the peaks at 778.0 and 780.4 eV were assigned to Co^3+^ and Co^2+^ of Co 2*p*_3/2_, whereas those at 793.8 and 797.2 eV were attributed to Co^3+^ and Co^2+^ of Co 2*p*_1/2_, respectively. The Se 3*d* spectrum of CoSe_2_‐NiSe_2_/NPFC exhibited four peaks: at 53.3 and 54.2 eV (Se 3*d*_5/2_) and 55.87 and 56.65 eV (Se 3*d*_3/2_) (Figure 3f). The Ni 2*p* peak of CoSe_2_‐NiSe_2_/NPFC was slightly redshifted compared with NiSe_2_/NPFC, whereas the Co 2*p* peak exhibited a blue shift compared with CoSe_2_/NPFC because Ni is more electronegative than Co. A blue shift of the Se peak was also observed in the heterostructured CoSe_2_‐NiSe_2_/NPFC compared to other samples, indicating a lower electron density around the Se atom. The above results suggest that CoSe_2_‐NiSe_2_/NPFC displays favorable electron transfer in the heterogeneous interface between NiSe_2_ and CoSe_2_ phases in close contact.

The doping of N, P, and F atoms was examined by high‐resolution XPS spectra of C 1*s* (Figure S6a, Supporting Information). Given that calcinated carbon does not have numerous oxygen‐containing functional groups, the doped atoms easily formed N–C (285.3 eV), P–C (285.7 eV), and F–C (288.3 eV) bonds. The N 1*s* spectrum exhibited peaks of pyridine‐N (398.4 eV), pyrrole‐N (398.6 eV), and graphite‐N (400.8 eV), thus indicating the presence of C–N bonds in NPFC (Figure S6b, Supporting Information). According to the XPS results discussed above, the MOF‐derived carbon was successfully doped with N, P, and F atoms when IL was used during synthesis. In contrast to the peak at 134.4 eV, which is compatible with a P–O bond, the P 2*p* peaks at 132.3 and 133.2 eV were assigned to 2*p*_1/2_ and 2*p*_3/2_ of P^3−^, respectively. When the metal phosphide was exposed to air, surface oxidation resulted in a phosphate signal (Figure S6c, Supporting Information). The P atom was successfully doped into the carbon produced from MOF as shown by the P–C bond (136.8 eV). Meanwhile, the F–C bond is responsible for the F 1*s* peak at 688.6 eV (Figure S6d, Supporting Information).

X‐ray absorption spectroscopy (XAS) was employed to further probe electronic state change in CoSe_2_‐NiSe_2_/NPFC due to heterostructure formation. The Ni K‐edge XAS spectra of NiSe_2_/NPFC and CoSe_2_‐NiSe_2_/NPFC are shown in Figure 3g.^[^ ^41^, ^42^, ^43^ ^]^ The latter exhibited a shift in the absorption threshold toward higher energies (inset of Figure 3g), thus indicating a higher electronic density over NiSe_2_ in the presence of heterostructures. Moreover, the Co K‐edge in CoSe_2_‐NiSe_2_/NPFC exhibited a shift in the opposite direction (Figure 3h). The above analysis indicates that the heterojunctions induced a redistribution of interfacial charge. Given that Ni is more electronegative than Co, electrons move toward NiSe_2_, which leads to a shift of the absorption threshold to higher energies.

To better understand the detailed coordination environment in CoSe_2_‐NiSe_2_/NPFC, we obtained the Fourier‐transformed (FT) EXAFS spectra of the Ni K‐edge of CoSe_2_‐NiSe_2_/NPFC and NiSe_2_/NPFC, as well as the Co K‐edge of CoSe_2_‐NiSe_2_/NPFC and CoSe_2_/NPFC (Figure 3i,j). The FT EXAFS spectrum of the Ni K‐edge for CoSe_2_‐NiSe_2_/NPFC and NiSe_2_/NPFC exhibited a dominant peak at around 2.47 Å that corresponds to the Ni–Se bond. In CoSe_2_‐NiSe_2_/NPFC and CoSe_2_/NPFC, the FT EXAFS spectrum of the Co K‐edge exhibited a dominant peak at around 2.4 Å, which corresponds to the Co–Se bond. Moreover, Ni and Co in CoSe_2_‐NiSe_2_/NPFC have lower coordination numbers compared with those in NiSe_2_/NPFC and CoSe_2_/NPFC, respectively (Table S1, Supporting Information), which may be due to the coexistence of heterogeneous spin states at the heterointerface and the mismatch in the degree of strong Jahn–Teller distortion. In the wavelet transformed (WT) EXAFS contour plots (Figure 3k,l), the significant shifts of Ni–Se and Co–Se maxima in CoSe_2_‐NiSe_2_/NPFC relative to NiSe_2_/NPFC and CoSe_2_/NPFC further demonstrate their different coordination environments and structural disorders.

### 2.2 The Test of HER

**Figure 4:**
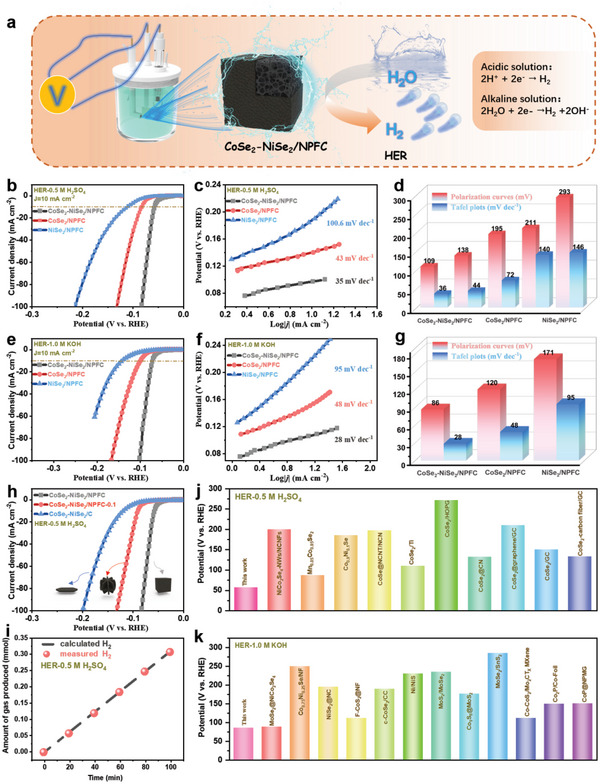
a) Illustration of the HER process. b) Polarization curves of CoSe_2_‐NiSe_2_/NPFC, CoSe_2_/NPFC, and NiSe_2_/NPFC in 0.5 M H_2_SO_4_; c) corresponding Tafel plots; and d) calculated electrochemical parameters. e) HER polarization curves of CoSe_2_‐NiSe_2_/NPFC, CoSe_2_/NPFC, and NiSe_2_/NPFC in 1.0 KOH; f) corresponding Tafel plots; and g) calculated electrochemical parameters. h) Linear scanning voltammetry (LSV) curves of CoSe_2_‐NiSe_2_/NPFC, CoSe_2_‐NiSe_2_/NPFC‐0.1, and CoSe_2_‐NiSe_2_/C. i) Measured and calculated amounts of gas produced over CoSe_2_‐NiSe_2_/NPFC at a current density of 10 mA cm^−2^. Comparison of the HER overpotential of CoSe_2_‐NiSe_2_/NPFC with reference electrocatalysts in (j) acidic and (k) alkaline solutions.

**Figure** **4**a shows the schematic setup and mechanism of HER. The electrocatalytic performance of CoSe_2_‐NiSe_2_/NPFC in HER was measured in a three‐electrode system in acidic and alkaline media using the prepared sample as the working electrode, a graphite rod as the counter electrode, and Ag/AgCl as the reference electrode. Figure 4b,e shows the linear scanning voltammetry (LSV) curves of CoSe_2_‐NiSe_2_/NPFC, CoSe_2_/NPFC, and NiSe_2_/NPFC at a scan rate of 5 mV s^−1^. Evidently, CoSe_2_‐NiSe_2_/NPFC exhibited excellent catalytic performance in both acidic and alkaline media as it required the respective overpotentials of only 57 and 86 mV to achieve a current density of 10 mA cm^−2^. Its performance was better than that of CoSe_2_/NPFC (*η*_j10_ = 82 mV in 0.5 M H_2_SO_4_; *η*_j10_ = 120 mV in 1.0 M KOH) and NiSe_2_/NPFC (*η*_j10_ = 111 mV in 0.5 M H_2_SO_4;_ *η*_j10_ = 171 mV in 1.0 M KOH), thus demonstrating the contribution of heterojunction to the HER performance.

The Tafel slopes of CoSe_2_‐NiSe_2_/NPFC, CoSe_2_/NPFC, and NiSe_2_/NPFC were calculated to elucidate the mechanism of HER. From Figure 4c,f, CoSe_2_‐NiSe_2_/NPFC displays a Tafel slope of 35 and 28 mV dec^−1^ in acidic and basic media, respectively. These values are superior to (i.e., smaller than) those of CoSe_2_/NPFC (43 mV dec^−1^ in 0.5 M H_2_SO_4_, 48 mV dec^−1^ in 1.0 M KOH) and NiSe_2_/NPFC (100.6 mV dec^−1^ in 0.5 M H_2_SO_4_, 95.0 mV dec^−1^ in 1.0 M KOH). The Tafel slopes obtained from LSV curves and Tafel plots are compared in Figure 4d (for acidic medium) and Figure 4g (for alkaline medium).

The LSV curves in Figure 4h demonstrated that CoSe_2_‐NiSe_2_/NPFC exhibited better performance than CoSe_2_‐NiSe_2_/NPFC‐0.1 and CoSe_2_‐NiSe_2_/C, thus demonstrating that the porous structure and heteroatom doping introduced by IL enhanced the HER performance. In addition, the Faraday efficiency of HER for CoSe_2_‐NiSe_2_/NPFC was close to 100%, thus indicating negligible side reactions as well as a high reaction efficiency in acidic (Figure 4i). Compared with previously reported highly active electrocatalysts, CoSe_2_‐NiSe_2_/NPFC exhibited significantly enhanced HER electrocatalytic activity (Figure 4j,k, and Tables S2 and S3, Supporting Information). In addition, constant‐potential electrochemical impedance spectroscopy (EIS) analysis further verified the enhanced interfacial properties of CoSe_2_‐NiSe_2_/NPFC under HER conditions (Figure S7a,b, Supporting Information).

**Figure 5:**
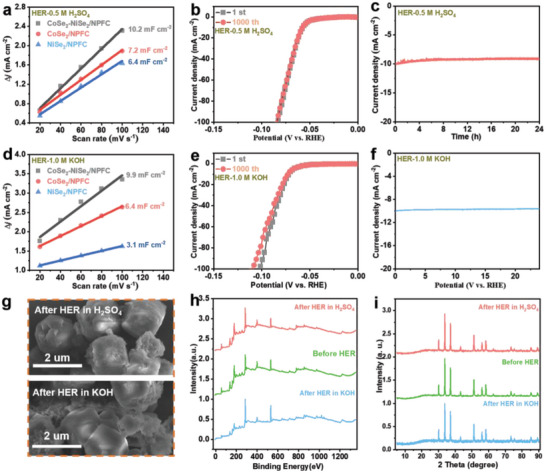
a) *C*_dl_ and normalized electrochemically active surface area of CoSe_2_‐NiSe_2_/NPFC and other references in 0.5 M H_2_SO_4_. b) Polarization curves of CoSe_2_‐NiSe_2_/NPFC before and after 1000 cyclic voltammetry (CV) cycles in 0.5 M H_2_SO_4_. c) Cycling stability of CoSe_2_‐NiSe_2_/NPFC after 24 h at −10 mA cm^−2^ in 0.5 M H_2_SO_4_. d) *C*_dl_ and normalized electrochemically active surface area of CoSe_2_‐NiSe_2_/NPFC and other references in 1.0 M KOH. e) Polarization curves of CoSe_2_‐NiSe_2_/NPFC before and after 1000 CV cycles in 1.0 M KOH. f) Cycling stability of CoSe_2_‐NiSe_2_/NPFC after 36 h at −10 mA cm^−2^ in 1.0 M KOH. g) SEM, h) XPS, and i) XRD results before and after HER in 0.5 M H_2_SO_4_ and 1.0 M KOH.

The HER activity of CoSe_2_‐NiSe_2_/NPFC was further investigated by measuring the double‐layer capacitance (*C*_dl_). Cyclic voltammetry (CV) curves of each catalyst were obtained in the potential range of 0.40–0.46 V at different scan rates (10–100 mV s^−1^) under acidic and alkaline conditions (Figures S8a–c–S10a,b). CoSe_2_‐NiSe_2_/NPFC exhibited the highest *C*_dl_ values (10.2 and 9.9 mF cm^−2^ in acidic and alkaline conditions, respectively), thus indicating abundant exposed electrocatalytic active sites therein (Figure 5a,d, and Figure S10c, Supporting Information).

The intrinsic HER catalytic activities of CoSe_2_‐NiSe_2_/NPFC, CoSe_2_/NPFC, and NiSe_2_/NPFC were further assessed under acidic and alkaline conditions using the double‐layer capacitance (C_dl_) and normalized electrochemically active surface area (ECSA) (Figure S11a,b, Supporting Information). CoSe_2_‐NiSe_2_/NPFC exhibited the highest HER performance according to the ECSA normalized geometric catalytic current density calculated from *C*_dl_: overpotentials of 24 and 51 mV at ECSA normalized catalytic current densities of 100 mA cm^−2^ in acidic and alkaline solutions, respectively.

The turnover frequency (TOF) is another important indicator of the internal activity of catalysts (Figure S12a,b, Supporting Information). After calculation, the TOF of CoSe_2_‐NiSe_2_/NPFC was 1.21 s^−1^, which stands for 1.21 molecules of H_2_ produced per second per active site, whereas the values of CoSe_2_/NPFC and NiSe_2_/NPFC were much lower (0.78 and 0.34 s^−1^, respectively). Therefore, CoSe_2_‐NiSe_2_/NPFC has higher activity than the two monometallic catalysts.

To test the stability of the material, the polarization curves of Ru/NBF‐NiSe_2_/Mo_2_CT*_x_* were measured after 1000 voltammetric tests in acidic and alkaline solutions, with no significant changes in performance (**Figure** **5**b,e). After 24 h of continuous cycling in 0.5 M H_2_SO_4_ and 1.0 M KOH, the chronoamperometry curves (Figure 5c,f) showed only a slight decrease. The stability of CoSe_2_‐NiSe_2_/NPFC was also investigated using SEM, XRD, and XPS analyses after 24 h HER tests in acidic and alkaline media. The surface morphology did not change significantly according to the SEM images (Figure 5g). Additionally, the XPS spectra (Figure 5h) and XRD patterns (Figure 5i) before and after the stability test were nearly identical. These results show that CoSe_2_‐NiSe_2_/NPFC was extremely stable during the HER.

### 2.3 The Test of Supercapacitor

**Figure 6:**
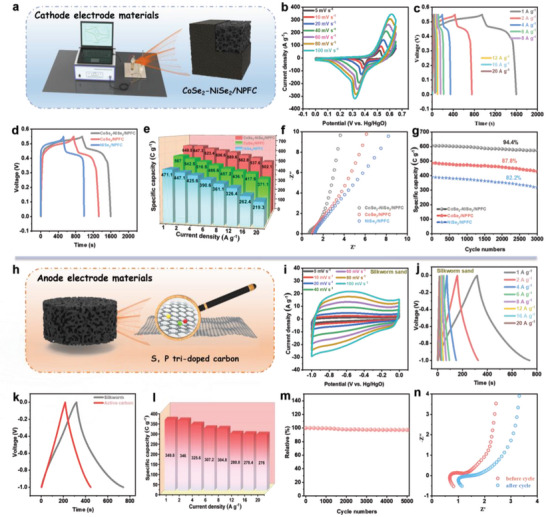
a) Process used to test the supercapacitor. b) CV and c) galvanostatic charge–discharge (GCD) curves of CoSe_2_‐NiSe_2_/NPFC electrode at different scan rates. d) CV, e) rate performance, f) EIS, and g) durability data of CoSe_2_‐NiSe_2_/NPFC, CoSe_2_/NPFC, and NiSe_2_/NPFC. h) Illustration of the S, P co‐doped carbon. i) CV, j) GCD curves, k) the GCD curves before and after cycling, l) rate performance, m) durability performance and n) EIS before and after cycling of S, P co‐doped carbon.

**Figure** **6**a illustrates the three‐electrode setup used for testing the supercapacitor cathode materials. Figure 6b and Figure S13a,b, Supporting Information display the CV curves of CoSe_2_‐NiSe_2_/NPFC, CoSe_2_/NPFC, and NiSe_2_/NPFC at sweep rates of 5–100 mV s^−1^. Evidently, the shape of these curves was well maintained at different sweep rates, which indicates that the materials exhibited good rate performance. Each CV curve exhibited several distinct redox peaks that represent the typical cell behavior. CoSe_2_‐NiSe_2_/NPFC exhibited the widest redox peaks and the largest integrated area under the peak compared with other electrode materials, thus indicating its highest specific capacity during redox.

The galvanostatic charge‐discharge (GCD) curves of CoSe_2_‐NiSe_2/_NPFC and other samples were measured at current densities of 1–20 A g^−1^ (Figure 6c and Figure S13c,d, Supporting Information). It is noted that the CoSe_2_‐NiSe_2_/NPFC electrode also exhibited a longer discharge time at 1.0 A g^−1^ than CoSe_2_/NPFC and NiSe_2_/NPFC, also indicating its maximum specific capacity (649.5 C g^−1^, Figure 6d). CoSe_2_‐NiSe_2_/NPFC had the highest specific capacity during the reaction owing to the interaction of CoSe_2_‐NiSe_2_ heterojunctions. Detailed data on the rate performance of each material are summarized in Figure 6e. CoSe_2_‐NiSe_2_/NPFC had the highest rate performance of 77.3% at a current density of 20 A g^−1^.

The EIS results further revealed that CoSe_2_‐NiSe_2_/NPFC had the lowest charge‐transfer resistance as well as ideal diffusion behavior for ions in the electrolyte (Figure 6f). After 3000 cycles at a current density of 6 A g^−1^, CoSe_2_‐NiSe_2_/NPFC retained 94.4% of the initial performance, which was higher than that of CoSe_2_/NPFC and NiSe_2_/NPFC (Figure 6g). Additionally, we measured the CV, GCD, rate performance, and durability of CoSe_2_‐NiSe_2_/C and CoSe_2_‐NiSe_2_/NPFC‐0.1 (Figure S14a—f, Supporting Information). The catalysts produced without or with only 0.1 ml of IL exhibited inferior specific capacity, rate performance, and stability compared with CoSe_2_‐NiSe_2_/NPFC, owing primarily to the lower levels of heteroatom doping and the reduced specific surface area.

To fabricate the supercapacitor device, a negative electrode was obtained by calcining silica sand with S, P co‐doped porous carbon (SPC), as schematically shown in Figure 6h. SEM observation confirmed that SPC had the structure of porous carbon (Figure S15a, Supporting Information). EDS and elemental mapping results also confirmed that SPC contained S and P elements (Figure S15b,c, Supporting Information), and carbon peaks are clearly visible in the XRD pattern (Figure S15d, Supporting Information). CV and GCD curves of SPC (Figure 6i,j, respectively) revealed that this material has a specific capacity of 349.8 C g^−1^ at a current density of 1 A g^−1^, which is higher than that of commercial activated carbon (Figure 6k). The CV and GCD of the commercial activated carbon are shown in Figure S16a,b, Supporting Information, respectively. At a high current density of 20 A g^−1^, SPC maintained 78.9% of its initial performance (Figure 6l). After 5000 charge‐discharge cycles, the capacity retention rate was 97.1% (Figure 6m). The impedance data before and after cycling are shown in Figure 6n.

In addition, we investigated the reaction kinetics of these electrode materials based on the CV curves at different sweep rates. The measured current (*i*) depends on the sweep rate (*v*) according to the following equations.

(1) $$i=i_{cap}+i_{diff}=\alpha v^{b}$$

**Figure 7:**
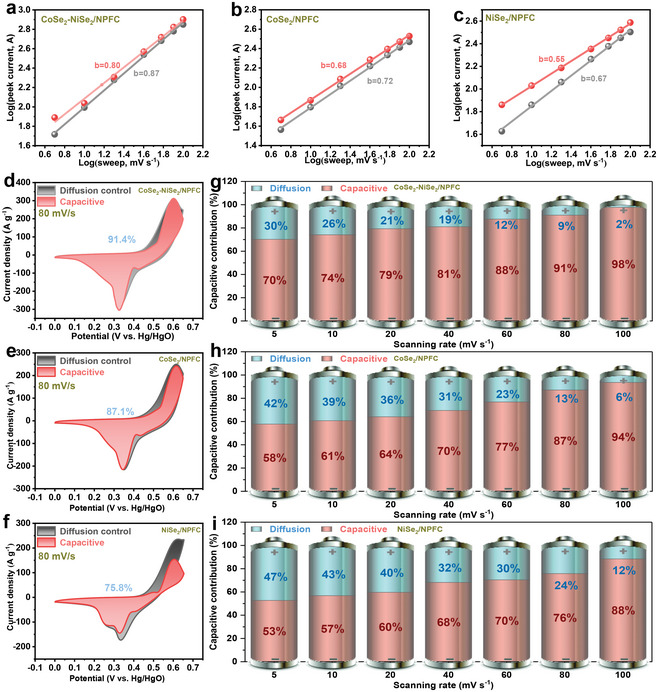
Log(i) versus log(v) plot for the oxidation and reduction peaks of a) CoSe_2_‐NiSe_2_/NPFC, b) CoSe_2_/NPFC, and c) NiSe_2_/NPFC. CV curves measured at 80 mV s^−1^ of d) CoSe_2_‐NiSe_2_/NPFC, e) CoSe_2_/NPFC, and f) NiSe_2_/NPFC showing the capacitive contribution (red) and diffusion‐controlled contribution (black). The ratio of capacitive and diffusion‐controlled contributions at different scan rates for g) CoSe_2_‐NiSe_2_/NPFC, h) CoSe_2_/NPFC, and i) NiSe_2_/NPFC.

(2) logi=logα+blogv

where *α* and *b* are empirical coefficients such that *b* = 0.5 implies that the electrochemical reaction is governed by diffusive behavior, *b* = 1.0 implies that the process is dominated by capacitive behavior, and *b* = 0.5–1.0 indicates a transition region between capacitive and battery‐type materials. From the corresponding plots for CoSe_2_‐NiSe_2_/NPFC, CoSe_2_/NPFC, and NiSe_2_/NPFC (**Figure** **7**a–c), CoSe_2_‐NiSe_2_/NPFC is more of a battery‐type material. Equations (3) and (4) described below could further quantitatively evaluate contributions from these two mechanisms to the total capacity at any scan rate. The capacitance and diffusion‐dominated capacities are represented by the first term (k_1_ *v*) and the second term (k_2_ *v* ^1/2^), respectively:

(3) $$i\left(V\right)=i_{cap}+i_{diff}=k_{1}v+k_{2}v^{1/2}$$

which can be simplified as

(4) $$i\left(V\right)v^{1/2}=k_{1}v^{1/2}+k_{2}$$

When *v* = 80 mV s^−1^, the capacitive contribution in CoSe_2_‐NiSe_2_/NPFC (91.4%) exceeded those in CoSe_2_/NPFC (87.1%) and NiSe_2_/NPFC (75.8%) (Figure 7d—f). All capacitance contributions of these electrodes at different scan rates are presented in Figures S17a–f–S19a—f, Supporting Information. The specific values are plotted in Figure 7g–i. Evidently, as the scan rate increased, the capacitive contribution increased, whereas the diffusion‐dominated fraction decreased. Furthermore, the capacitive contribution in CoSe_2_‐NiSe_2_/NPFC exceeded that in other electrodes at any scan rate, which is in excellent agreement with the qualitative analysis above based on the *b* value. These experimental results fully reveal the reason for the excellent specific capacity and optimum rate capability of the CoSe_2_‐NiSe_2_/NPFC electrode.

### 2.4 The Analysis of DFT

**Figure 8:**
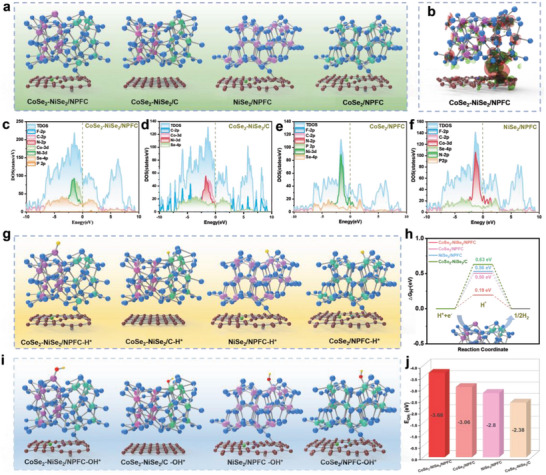
a) Ball‐and‐stick models of CoSe_2_‐NiSe_2_/NPFC, CoSe_2_‐NiSe_2_/C, CoSe_2_/NPFC, and NiSe_2_/NPFC. b) Electron density difference maps of CoSe_2_‐NiSe_2_/NPFC. The total density of states (TDOS) of c) CoSe_2_‐NiSe_2_/NPFC, d) CoSe_2_‐NiSe_2_/C, e) CoSe_2_/NPFC, and f) NiSe_2_/NPFC. g) Ball‐and‐stick models of the four materials in (a) after H^*^ adsorption. h) The *∆G*_H*_ of CoSe_2_‐NiSe_2_/NPFC, CoSe_2_‐NiSe_2_/C, CoSe_2_/NPFC, and NiSe_2_/NPFC. i) Ball‐and‐stick models of the four materials in (a) after OH^*^ adsorption. j) *E*_OH‐_ values of CoSe_2_‐NiSe_2_/NPFC, CoSe_2_/NPFC, NiSe_2_/NPFC, and CoSe_2_‐NiSe_2_/C.

DFT calculations were performed (for details see Supporting Information) to investigate the relationship between electrocatalytic performance and local coordination structures. **Figure** **8**a shows the constructed 3D models of CoSe_2_‐NiSe_2_/NPFC, CoSe_2_‐NiSe_2_/C, CoSe_2_/NPFC, and NiSe_2_/NPFC. The atoms represented by each ball are explained in Figure S20, Supporting Information. From the 3D isosurface of electrical charges (Figure 8b), the combination of CoSe_2_ with NiSe_2_ re‐distributes the charges, thereby providing a fast path for electron transmission and increasing H^*^ adsorption. Furthermore, according to the calculated density of states (DOS) profiles, CoSe_2_‐NiSe_2_/NPFC has a higher DOS near the Fermi level compared to the other three materials (CoSe_2_‐NiSe_2_/C, CoSe_2_/NPFC, and NiSe_2_/NPFC), thus implying that heterojunction formation and the doping of N, P, and F lead to stronger adsorption and faster electron transfer pathways (Figure 8c–f).

Next, the free energy of H^*^ adsorption was modeled on different materials (Figure 8g).

We first assessed the active sites on the NPF‐doped carbon (NPFC/CoSe_2_‐NiSe_2_) and on the CoSe_2_‐NiSe_2_, which shows that CoSe_2_‐NiSe_2_/NPFC performs better than NPFC/CoSe_2_‐NiSe_2_ (Figure S21a,b, Supporting Information)_._ Then compared with the H^*^ adsorption free energies (*∆G*_H*_) on CoSe_2_‐NiSe_2_/C (0.50 eV), CoSe_2_/NPFC (0.56 eV), and NiSe_2_/NPFC (0.63 eV), CoSe_2_‐NiSe_2_/NPFC exhibited the smallest *∆G*_H*_ of 0.19 eV (Figure 8h). This observation suggests that CoSe_2_‐NiSe_2_/NPFC exhibits the optimal H^*^ adsorption and desorption behaviors, which is consistent with the experimental results.

Furthermore, we calculated the free energy of OH^*^ adsorption on NiSe_2_/NPFC, CoSe_2_/NPFC, and NiSe_2_‐CoSe_2_/NPFC, and the results are summarized in Figure 8i,j. The heterogeneous structure of CoSe_2_‐NiSe_2_/NPFC resulted in much lower free energy of OH^*^ adsorption (−3.68 eV) compared with that of CoSe_2_/NPFC and NiSe_2_/NPFC, further demonstrating that the heterogeneous interface promotes OH^*^ adsorption, which also agrees with the experimentally observed fast reaction kinetics in the supercapacitor.

### 2.5 The Test of Flexible Supercapacitor

**Figure 9:**
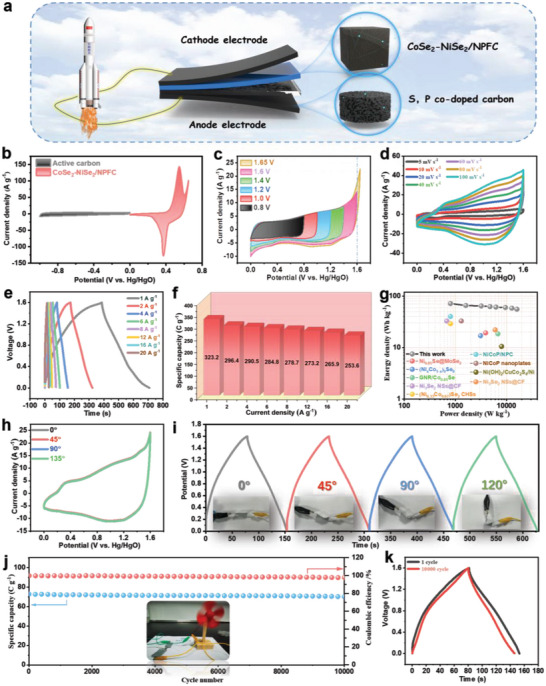
a) Structure of the as‐assembled flexible device with the configuration of CoSe_2_‐NiSe_2_/NPFC//SPC. b) CV curves of CoSe_2_‐NiSe_2_/NPFC and SPC at 20 mV s^−1^. c) CV curves of CoSe_2_‐NiSe_2_/NPFC at various potentials. d) CV curves of CoSe_2_‐NiSe_2_/NPFC//SPC at various scan rates. e) GCD curves collected at different current densities. f) Specific capacitance at different current densities. g) Ragone plots of the present device and previously reported ones. h) CV curves at a scan rate of 40 mV s^−1^ and i) GCD curves at a current density of 4 A g^−1^ for CoSe_2_‐NiSe_2_/NPFC//SPC, both collected during bending. j) Cycling performance and Coulombic efficiency of CoSe_2_‐NiSe_2_/NPFC//SPC at a current density of 4 A g^−1^. Inset: A fan powered by the flexible asymmetric supercapacitor device. k) GCD curves of CoSe_2_‐NiSe_2_/NPFC//SPC before and after cycling.

Flexible solid‐state supercapacitors are promising energy storage devices with various advantages, such as improved safety, excellent reliability, and easy scalability. To further demonstrate the benefits of CoSe_2_‐NiSe_2_/NPFC, a solid‐state flexible supercapacitor system was assembled using CoSe_2_‐NiSe_2_/NPFC cathode and SPC anode (**Figure** **9**a). First, the CV curves of the cathode and anode materials were collected at a current density of 20 A g^−1^ to determine a stable electrochemical window (Figure 9b). Next, CV curves were collected in different voltage ranges (0–1.7 V) to determine the appropriate voltage window. The CV curve shows polarization when the voltage reaches 1.7 V; therefore, 1.6 V was considered the most suitable voltage (Figure 9c). When CV tests were performed at different sweep rates, the curve shape was well maintained, whereas the current density values expanded gradually, thus implying a high rate performance (Figure 9d). The GCD curves were also measured at different current densities, and the specific capacity was determined to be 323.2 C g^−1^ at a current density of 1 A g^−1^ (Figure 9e). Finally, Figure 9f shows that the supercapacitor has excellent rate performance, maintaining 78.4% of its specific capacity at a current density of 20 A g^−1^.

In the Ragone plot (Figure 9g), the designed supercapacitor achieved an energy density of 55.7 Wh kg^−1^ at a power density of 15.96 kW kg^−1^, and an energy density of 71.8 Wh kg^−1^ at an average power density of 0.799 kW kg^−1^. The energy density and power density are better than other related transition metal compounds (Table S4, Supporting Information). The performance of the flexible supercapacitor was examined by recording its CV and GCD curves while bending it to the angles of 0°, 45°, 90°, and 120°. The bent supercapacitor exhibited virtually no degradation in performance, thereby confirming its structural integrity and flexibility (Figure 9h,i). Both the performance and Coulomb efficiency remained at 96.5% after 10000 charge‐discharge cycles, thus demonstrating the material's high cycling stability (Figure 9j). Figure 9k and Figure S22, Supporting Information displays the GCD and EIS data of CoSe_2_‐NiSe_2_/NPFC//SPC before and after cycling. EDS results of CoSe_2_‐NiSe_2_/NPFC after cycling are displayed in Figure S23, Supporting Information. From the XRD after cycling, The NiSe_2_ and CoSe_2_ are reacted with OH^–^ to form NiSeO_3_⋅H_2_O and CoSeO_4_⋅6H_2_O (Figure S24, Supporting Information).^[^ ^34^ ^]^

## 3 Conclusion

In summary, we have synthesized a series of Co‐Ni‐based MOFs with different morphologies by varying the volume of injected IL. When using a higher IL volume, the prepared CoSe_2_‐NiSe_2_/NPFC with a porous cube morphology exhibited a larger specific surface area nearly five times and more heteroatoms doping than that of the lamellar hollow structure of CoSe_2_‐NiSe_2_/C (synthesized without adding IL). Additionally, heterojunctions and heteroatoms introduced by IL affected the interfacial electronic structure, which in turn lowered the OH^*^/H^*^ adsorption energies. These changes are responsible for the fast reaction kinetics CoSe_2_‐NiSe_2_/NPFC, as confirmed by detailed experimental characterization and DFT calculations. CoSe_2_‐NiSe_2_/NPFC showed excellent catalytic performance during HER in both acidic and alkaline solutions, requiring a relatively lower overpotential of only 57 and 86 mV to achieve a current density of 10 mA cm^−2^, respectively. When used as the cathode in supercapacitors, the CoSe_2_‐NiSe_2_/NPFC electrode also sustained a longer discharge time (specific capacity: 649.5 C g^−1^) than that of other samples at the current density of 1.0 A g^−1^. This proposed design not only demonstrates a facile method to modulate MOF morphology using ILs but also provides new ideas for designing a complex of heterojunctions and heteroatom‐doped carbon derived from MOFs.

## 4 Experimental Section

### Synthesis of Co‐Ni‐MOF‐0, −0.1, and −0.2

Ni(NO_3_)_2_⋅6H_2_O (0.097 g, 0.5 mmol) and Co(NO_3_)_2_⋅6H_2_O (0.194 g, 1 mmol) were dissolved in 20 ml of deionized (DI) water and 0, 0.1, or 0.2 ml of 1‐hexyl‐3‐methylimidazole hexafluorophosphate IL, followed by stirring for 5 min. Separately, 0.65 g of dimethylimidazole was dissolved in the solution and mixed for 5 min. Subsequently, the prepared dimethylimidazole solution was added slowly into the metal precursor solution with a rubber‐tipped dropper under vigorous stirring and left to stand for 60 min. Next, the product was washed six times with H_2_O and CH_3_CH_2_OH alternately and further dried at 60 °C to obtain the Co‐Ni‐MOF precursor with a heterogeneous structure. These were labeled as Co‐Ni‐MOF‐0, −0.1, and −0.2 according to the amount of IL used in synthesis.

### Synthesis of CoSe_2_‐NiSe_2_/C, CoSe_2_‐NiSe_2_/NPFC‐0.1, and CoSe_2_‐NiSe_2_/NPFC

The dried Co‐Ni‐MOF precursors were mixed with 800 mg of Se powder in a mortar, and then heated to 400 °C in a tube furnace under an Ar/H_2_ atmosphere at a heating rate of 2 °C min^−1^ and held at this temperature for 2 h. This produced CoSe_2_‐NiSe_2_/NPFC. CoSe_2_/NPFC and NiSe_2_/NPFC samples were synthesized in a similar way.

### Characterization

XRD was performed on a TD‐3500 (Tongda, China) diffractometer, and SEM observations were made on a Hitachi SU‐8220 device. TEM images were captured with a JEM‐2100HR microscope (JEOL, Japan). XPS data were measured using a K‐Alpha+ photoelectron spectrometer (Thermo Fisher Scientific).

### Electrochemical Evaluations

Electrochemical tests to evaluate the HER performance were performed in a three‐electrode system with an acidic or basic electrolyte (0.5 M H_2_SO_4_ or 1.0 M KOH, respectively), Ag/AgCl reference electrode, and graphite rod as the counter electrode. A catalyst ink was prepared by dispersing a 10 mg sample in 1 ml mixed liquid (0.75 ml of water, 0.15 ml of ethyl alcohol, and 0.1 ml of 5 wt% Nafion) and sonicating for 2 h. Next, a micro syringe was used to drop 8 µl of the ink onto a glassy carbon (GC) electrode, followed by drying at room temperature. LSV was performed in 0.5 M H_2_SO_4_ at room temperature and with a scanning rate of 5 mV s^−1^. All measured potentials were converted to the reversible hydrogen electrode (RHE): E (RHE) = E (Hg/Hg_2_Cl_2_) + (0.242 + 0.059 × pH) in a unit of *V*. The Tafel slopes were calculated by plotting the overpotential against log |j| from the LSV data.

To prepare the supercapacitors, the catalyst (CoSe_2_‐NiSe_2_/NPFC), carbon black, and polyvinylidene fluoride (PVDF) were mixed in a mass ratio of 7:2:1 and prepared into a slurry with N‐methylpyrrolidone (NMP). Subsequently, the resulting slurry was covered over a nickel foam as the working electrode. To determine the mass of the loaded active ingredient, the working electrode was pressed at 10 MPa and 60 °C for 24 h. All electrochemical experiments related to the supercapacitor were conducted in a 3.0 M KOH solution, with platinum foil as the counter electrode and Hg/HgO as the reference electrode. An electrochemical workstation (CHI760e, CH Instruments) was used for CV, EIS, and GCD measurements.

## Conflict of Interest

The authors declare no conflict of interest.

## Supporting information

Supporting Information

## Data Availability

The data that support the findings of this study are available from the corresponding author upon reasonable request.
