# Supplementary material for: Ionic Liquid Meets MOF: A Facile Method to Optimize the Structure of CoSe2‐NiSe2 Heterojunctions with N, P, and F Triple‐Doped Carbon Using Ionic Liquid for Efficient Hydrogen Evolution and Flexible Supercapacitors
Source: Adv Sci (Weinh). 2023 Jan 13;10(7):2206029. doi: 10.1002/advs.202206029 (PMC9982578; doi:10.1002/advs.202206029)
Supplement: Supplementary file 1 — Supporting Information [file ADVS-10-2206029-s001.pdf]

ADVANCED SCIENCE
Open Access

Supporting Information

for *Adv. Sci.*, DOI 10.1002/advs.202206029

Ionic Liquid Meets MOF: A Facile Method to Optimize the Structure of CoSe2-NiSe2 Heterojunctions with N, P, and F Triple-Doped Carbon Using Ionic Liquid for Efficient Hydrogen Evolution and Flexible Supercapacitors

Mingjie Yi, Jiayu Ma, Yi Ren, Hao Wang, Lin Xie, Zhenye Zhu and Jiaheng Zhang**

Ionic Liquid Meets MOF: A Facile Method to Optimise the Structure of $CoSe_2$-$NiSe_2$ Heterojunctions with N, P, and F Triple-Doped Carbon Using Ionic Liquid for Efficient Hydrogen Evolution and Flexible Supercapacitors

Mingjie Yi, Jiayu Ma, Yi Ren, Hao Wang, Lin Xie, Zhenye Zhu, Jiaheng Zhang

M. J. Yi, J. Y. Ma, Y. Re, H. Wang, L. Xie, Prof. Z. Y. Zhu, Prof. J. H. Zhang

State Key Laboratory of Advanced Welding and Joining, Harbin Institute of Technology, Shenzhen 518055, Guangdong, China.

Email: zhangjiaheng@hit.edu.cn; zhuzy@hit.edu.cn

M. J. Yi, J. Y. Ma, Y. Re, H. Wang, L. Xie, Prof. Z. Y. Zhu, Prof. J. H. Zhang

Research Centre of Printed Flexible Electronics, School of Materials Science and Engineering, Harbin Institute of Technology, Shenzhen 518055, Guangdong, China.

Email: zhangjiaheng@hit.edu.cn; zhuzy@hit.edu.cn

Density functional theory calculations:

For all density functional theory (DFT) calculations, we employed the Vienna ab initio simulation package (VASP) [1,2] within the generalised gradient approximation (GGA) using the Perdew–Burke–Ernzerhof (PBE) formulation [3]. We chose the projected augmented wave (PAW) potentials [4,5] to describe the ionic cores. The valence electrons were taken into account using a plane wave basis set with a kinetic energy cutoff of 400 eV. Partial occupancies of the Kohn-Sham orbitals were allowed using the Gaussian smearing method and a width of 0.05 eV. The electronic energy was considered self-consistent when the energy change was smaller than 10^{-5} eV. Geometry optimisation was considered convergent when the force change was smaller than 0.05 eV/Å. Grimme's DFT-D3 methodology [6] was used to describe the dispersion interactions. The Brillourin zone was sampled with a gamma-centred 2 × 2 × 1 grid in all computational processes [7].

The adsorption energy of OH^- (E_{ads}) was calculated as $E_{ads} = E_{total} - E_{slab} - E_{OH-}$, where E_{total} and E_{slab} represent the energy with and without the OH^- of slab, and E_{OH-} is the energy of an OH^- ion.

Calculation of free energies

The adsorption free energies of H atom on all structures were calculated by the formula $\Delta G^*_H = \Delta E^*_H + \Delta ZPE - T\Delta S$, where ΔE^*_H, ΔZPE, and ΔS are the binding energy, zero-point energy change, and entropy change of H adsorption, respectively. The entropies and zero-point energies (ZPEs) of H_2 and H^* came from previous reports [8, 9].

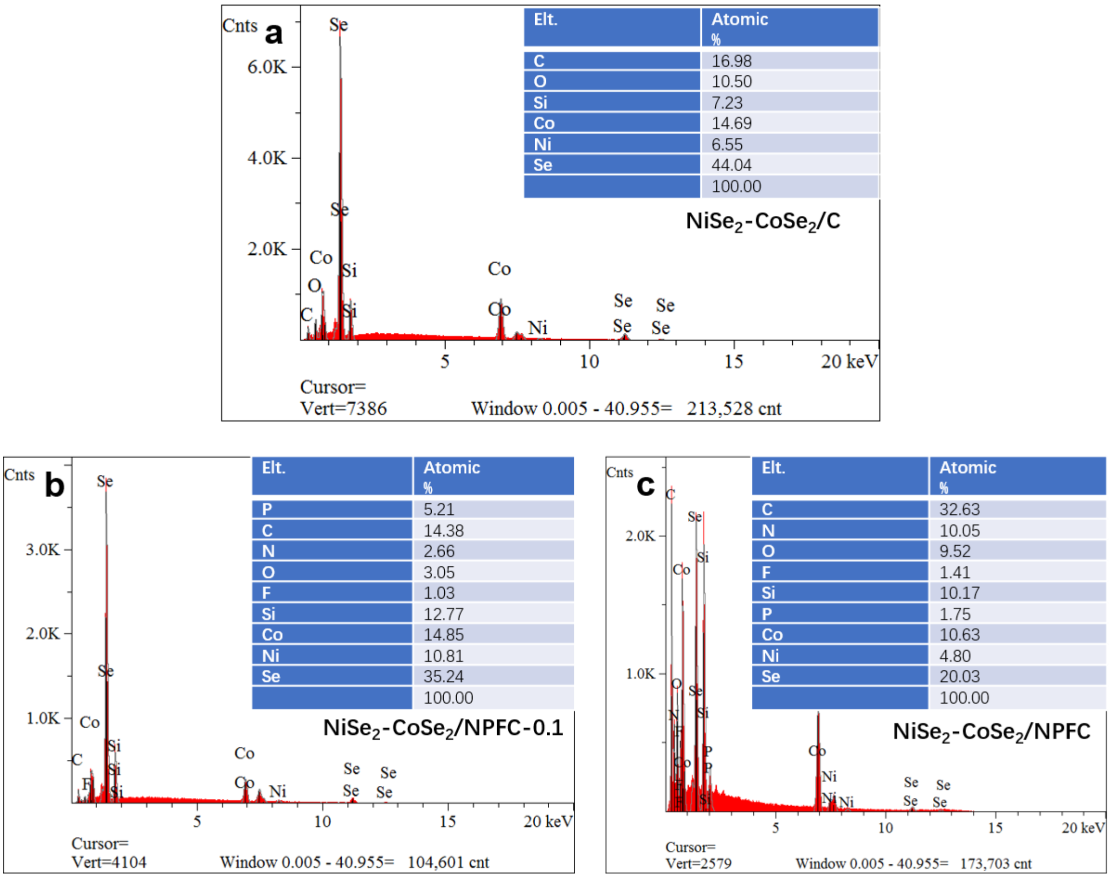

Figure S1. The EDS of (a) $CoSe_2$-$NiSe_2$/C, (b) $CoSe_2$-$NiSe_2$/NPFC-0.1, and (c) $CoSe_2$-$NiSe_2$/NPFC.

。

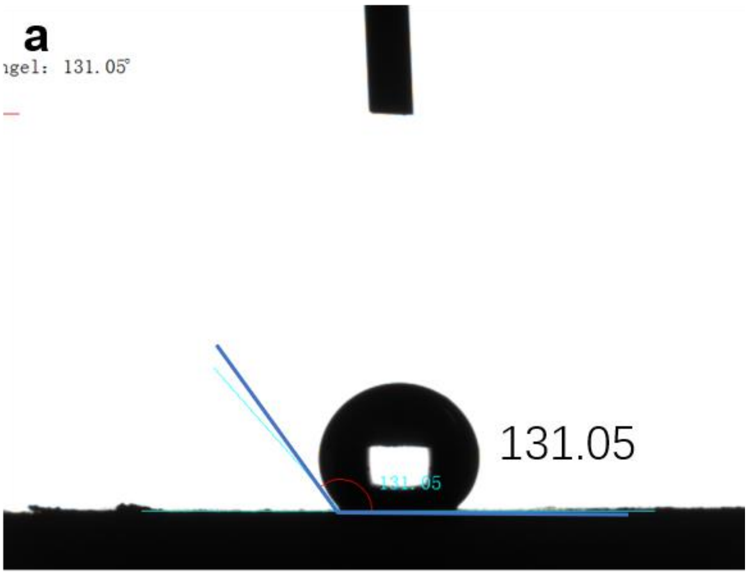

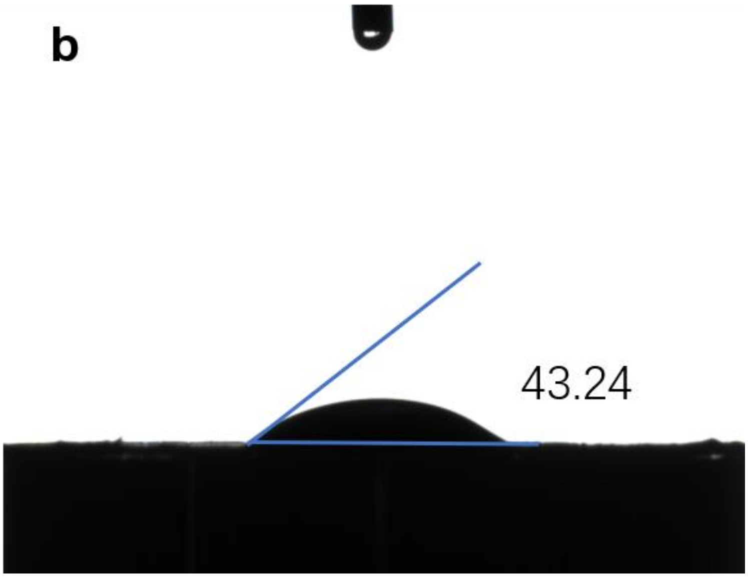

Figure S2. Images above and below show the water contact angles of (a) $CoSe_2$-$NiSe_2$/C, (b) $CoSe_2$-$NiSe_2$/NPFC

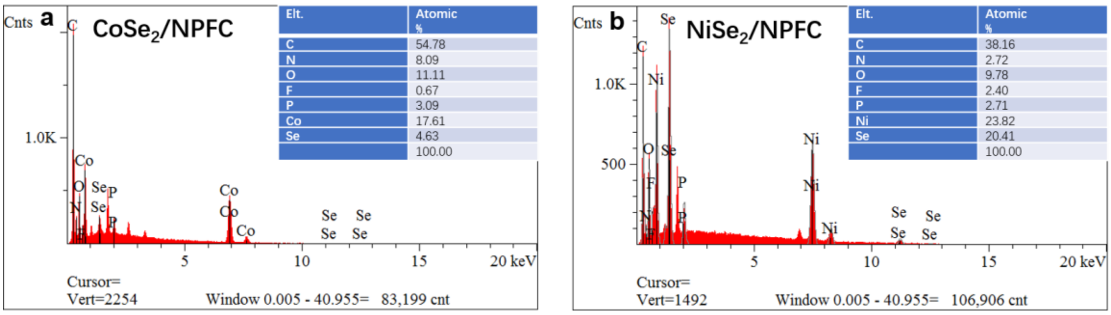

Figure S3. The EDS of (a) $CoSe_2$-$NiSe_2$/NPFC, (b) $NiSe_2$/NPFC.

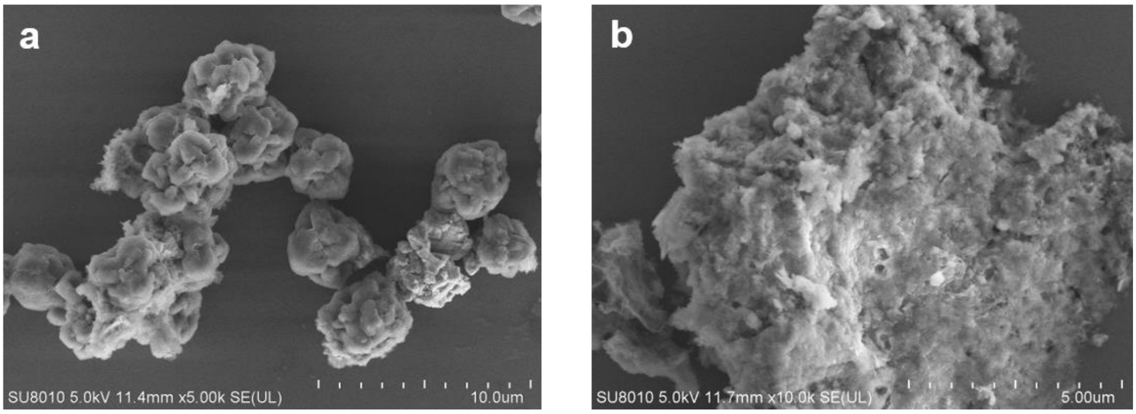

Figure S4. The SEM of (a) $CoSe_2$ /NPFC, (b) $NiSe_2$/NPFC.

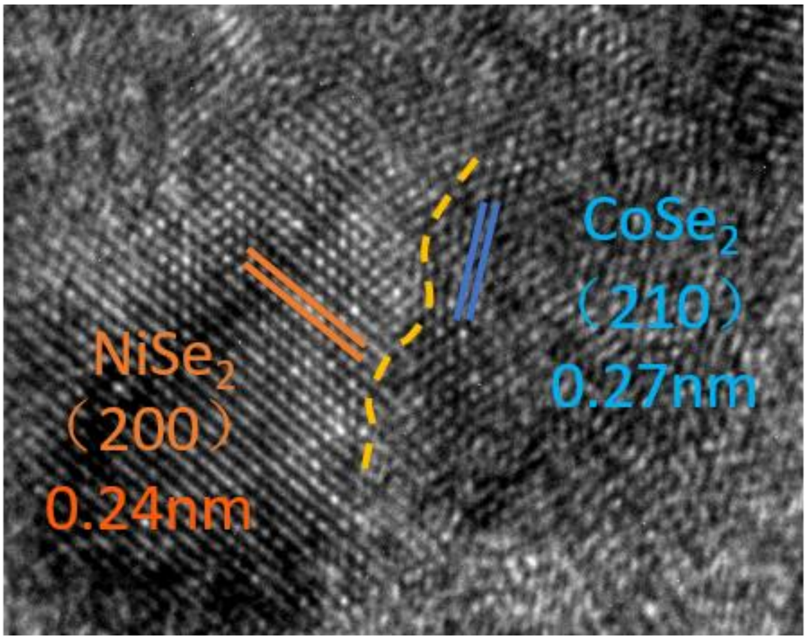

Figure S5. High-resolution TEM image of (a) $CoSe_2$-$NiSe_2$/NPFC.

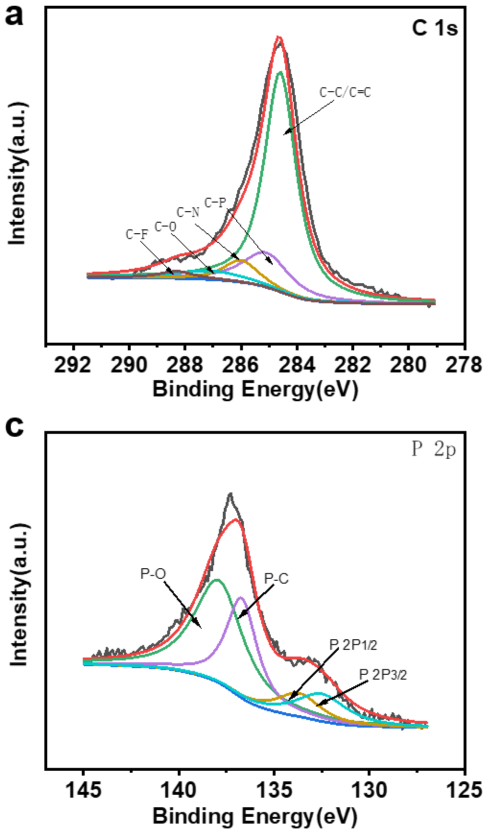

Figure S6. The XPS of (a) N 1s, (b)P 2p, (c)F 1s, and (d)C 1s.

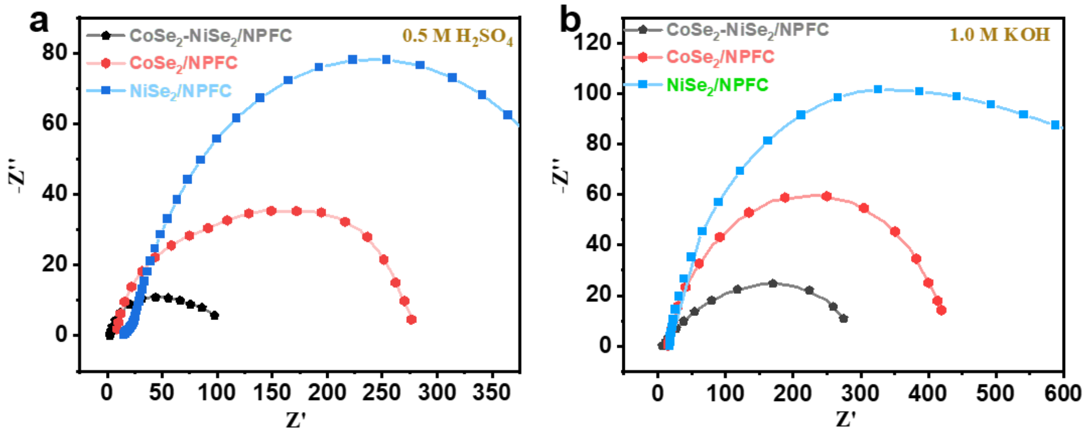

Figure S7. EIS of $CoSe_2$-$NiSe_2$/NPFC, $CoSe_2$/NPFC and $NiSe_2$/NPFC in (a) 0.5 H_2SO_4 and (b) 1.0 M KOH.

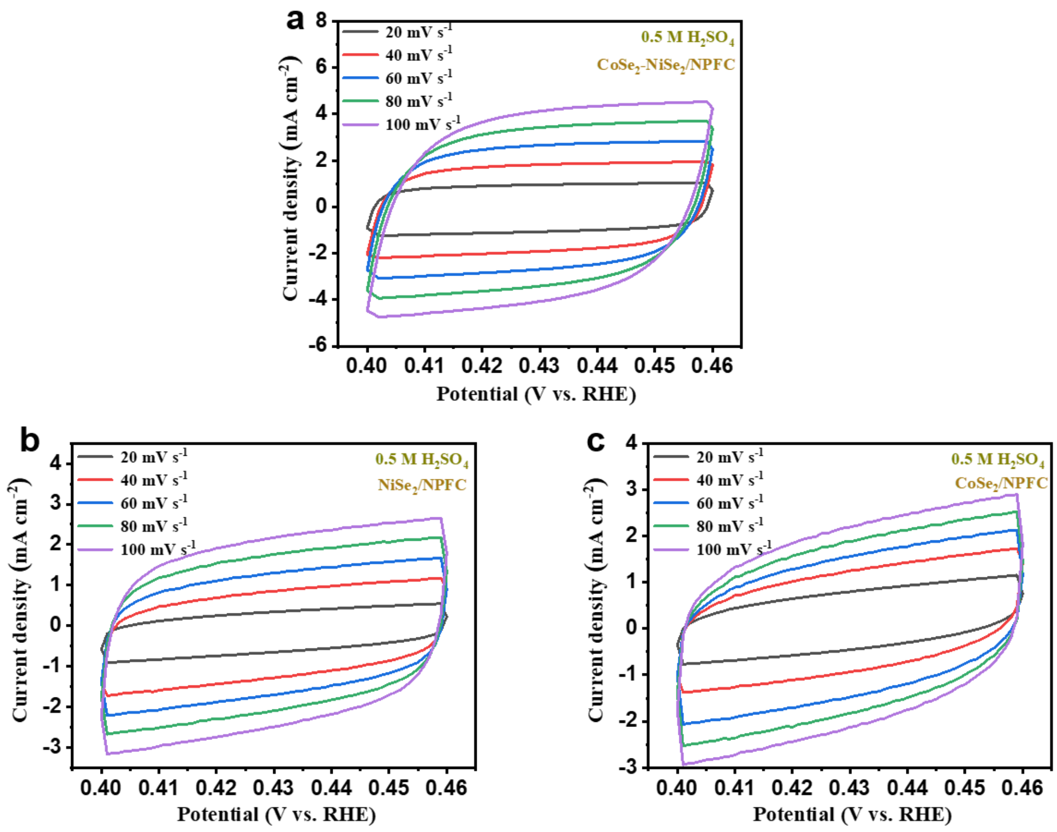

Figure S8. Oltammograms of (a) $CoSe_2$-$NiSe_2$/NPFC, (b) $CoSe_2$/NPFC and (c) $NiSe_2$/NPFC. at various scan rates (10–100 mV s^{-1}) used to estimate the Cdl and relative electrochemically active surface area in 0.5 M H_2SO_4.

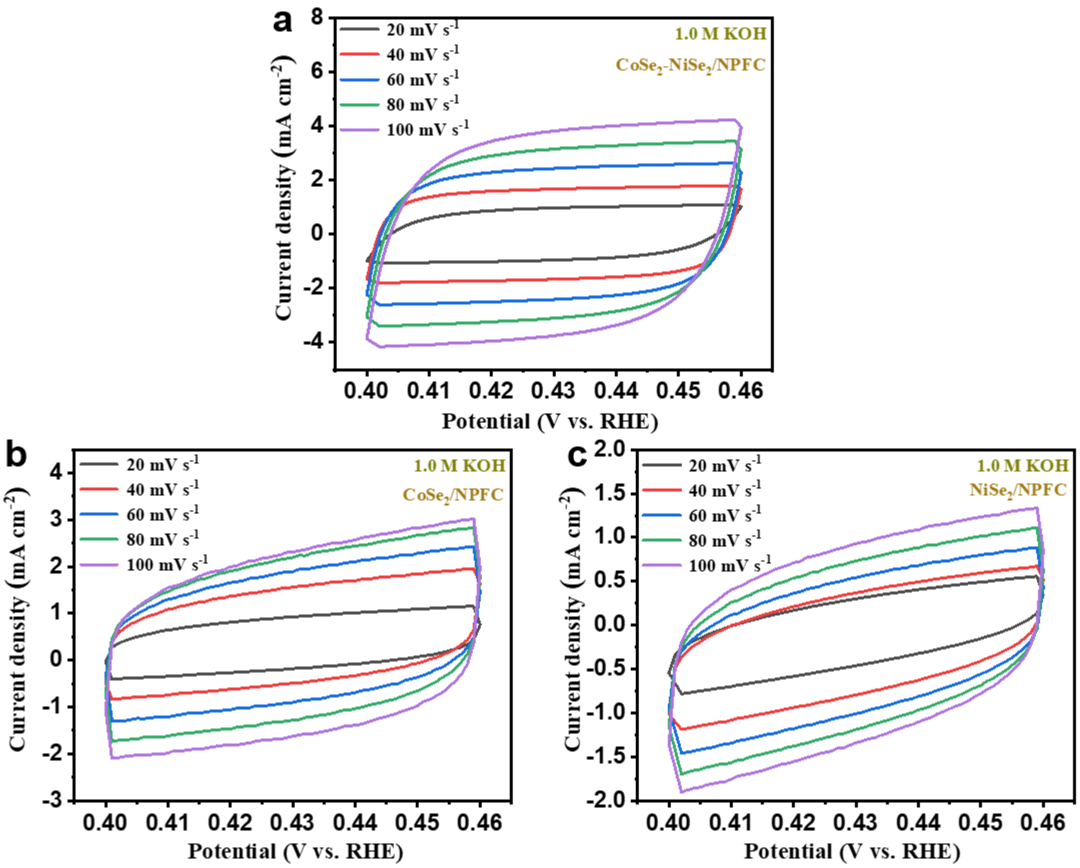

Figure S9. Oltammograms of (a) $CoSe_2$-$NiSe_2$/NPFC, (b) $CoSe_2$/NPFC and (c) $NiSe_2$/NPFC. at various scan rates (10–100 mV s^{-1}) used to estimate the Cdl and relative electrochemically active surface area in 0.5 M 1.0 KOH.

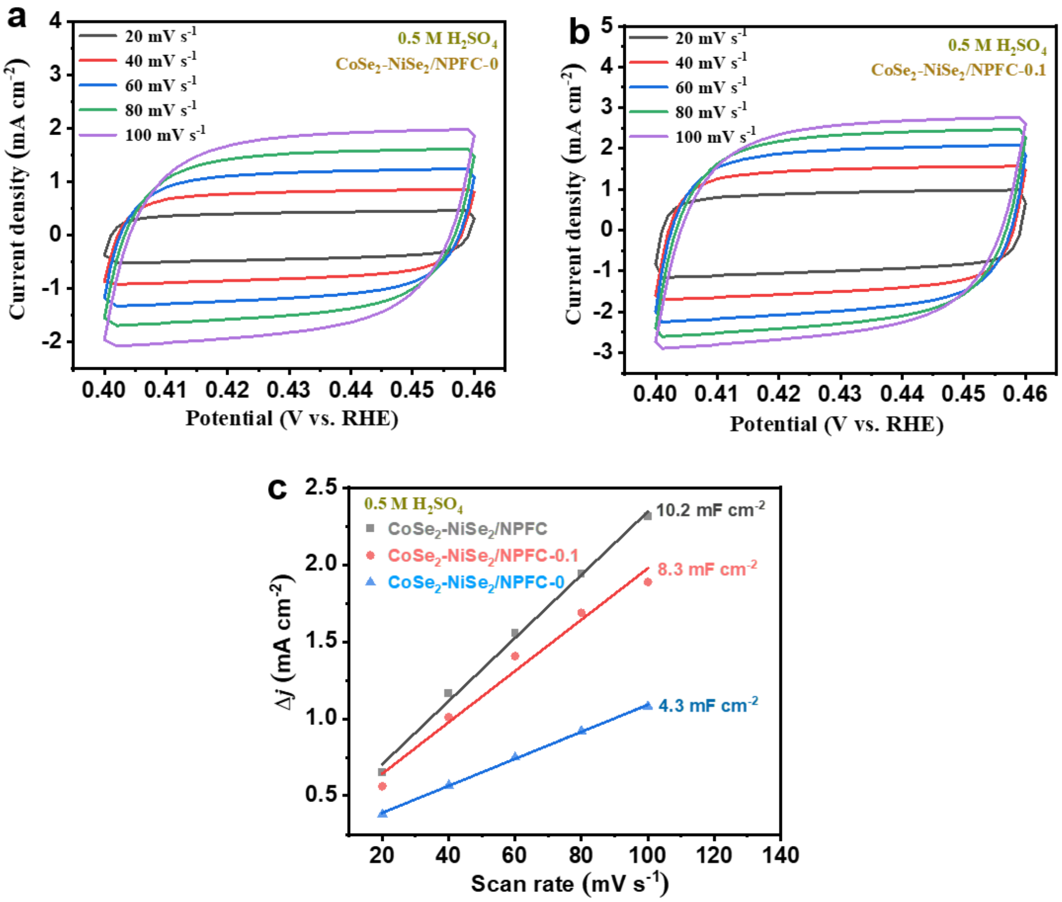

Figure S10. Oltammograms of (a) $CoSe_2$-$NiSe_2$/NPFC-0 and (b) $CoSe_2$-$NiSe_2$/NPFC-0.1. at various scan rates (10–100 mV s^{-1}) used to estimate the Cdl and relative electrochemically active surface area in 0.5 M H_2SO_4. (c) Cdl and relative electrochemically active surface area of $CoSe_2$-$NiSe_2$/NPFC.

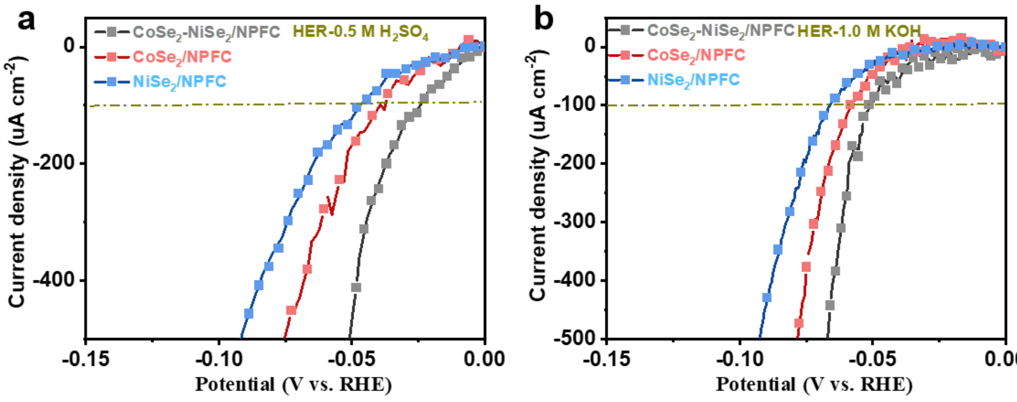

Figure S11. LSV curves of HER for $CoSe_2$-$NiSe_2$/NPFC, $CoSe_2$/NPFC and $NiSe_2$/NPFC normalized with ECSA in an (a) acidic solution and (b) alkaline solution.

Calculation of TOF

By using the previous reported method to calculate the active sites and TOF of $CoSe_2$-$NiSe_2$/NPFC (Adv. Energy Mater. 2018, 1800484), for all samples in our work, the activesites and TOF were counted through the following calculation (Power Sources 2015, 297, 45; Small 2018, 14, 1706237;).

In order to calculate the active sites for each catalyst, CV measurements with potential window from 0 V to 0.8 V were carried out in phosphate buffered saline (PBS, PH= 7), where we assumed that no hydrogen evolution reaction together with electrochemical corrosion of our samples happened. After plotting the potential divide the scan rate (20 mV) against the current density, the current density-time curves were obtained (Figure S12a). The absolute current density (I_1, A cm^{-2}) was integrated each second (s) to obtain the area (quantity of electric charge, Q) inside one cyclic envelope (A cm^{-2}×s= C cm^{-2}) containing both the catholic and anodic current density. In this window, reversible one electron redox was completed (one for reduction and another for oxidation). So, the active sites (n) were acquired by dividing Q with 2 and the Faradaic constant (F, 96485 C·mol^{-1}).

$$Q=\int_{40}^{0} I_1 dt$$

$$n=\frac{Q}{2F}$$

The TOF could be calculated with the following equation

$$TOF=\frac{I_2}{2nF}$$

Where I_2 was the current density from the LSV test in Figure 3, n was the obtained amount of the active site, and F was the Faradaic constant. For fare comparison, we selected the overpotential of 150 mV in acid for each specimen to get the value of I_2.

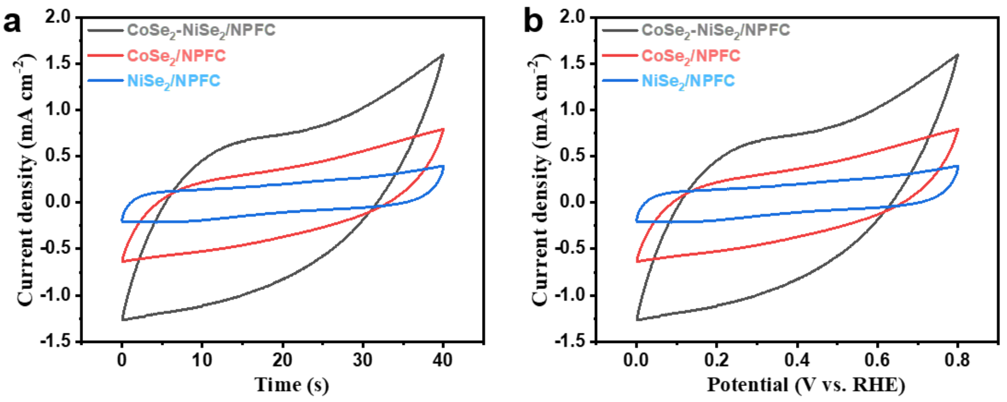

Figure S12. (a) CV curves of $CoSe_2$-$NiSe_2$/NPFC, $CoSe_2$/NPFC and $NiSe_2$/NPFC in 0.5 M H_2SO_4 and (b) corresponding current density-time curves.

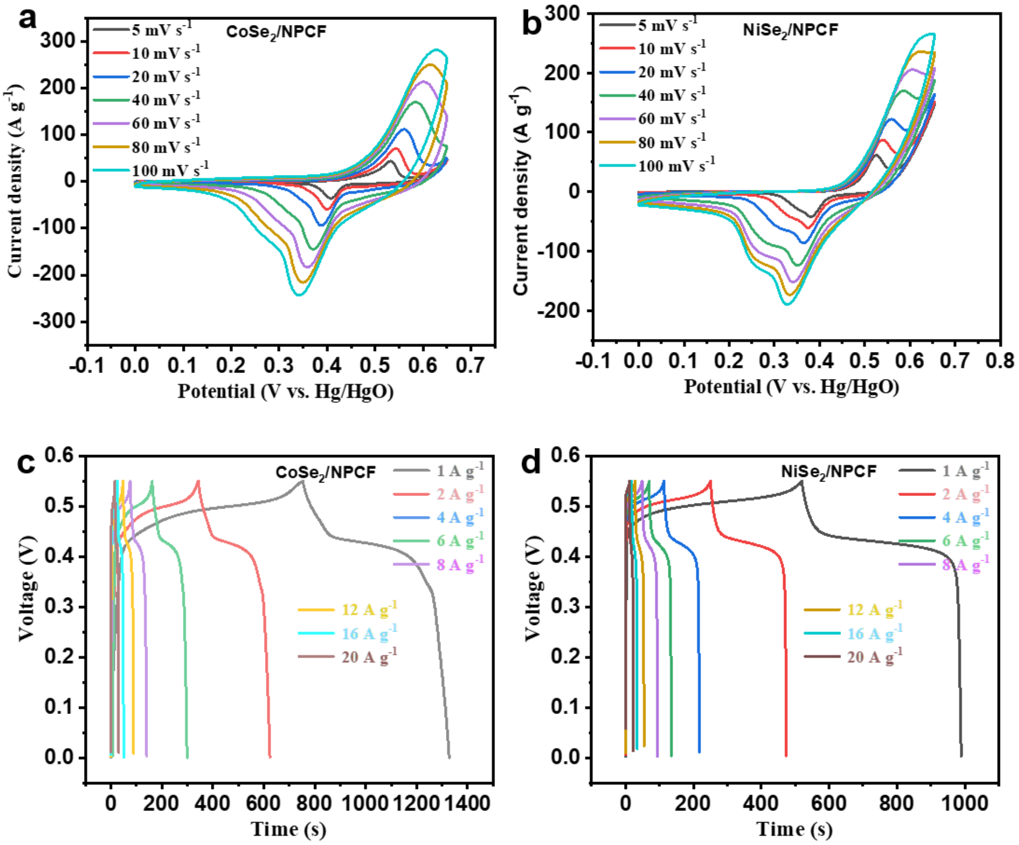

Figure S13. The CV of (a) $CoSe_2$/NPFC and (b) $NiSe_2$/NPFC; The GCD of (c) $CoSe_2$/NPFC and (d) $NiSe_2$/NPFC.

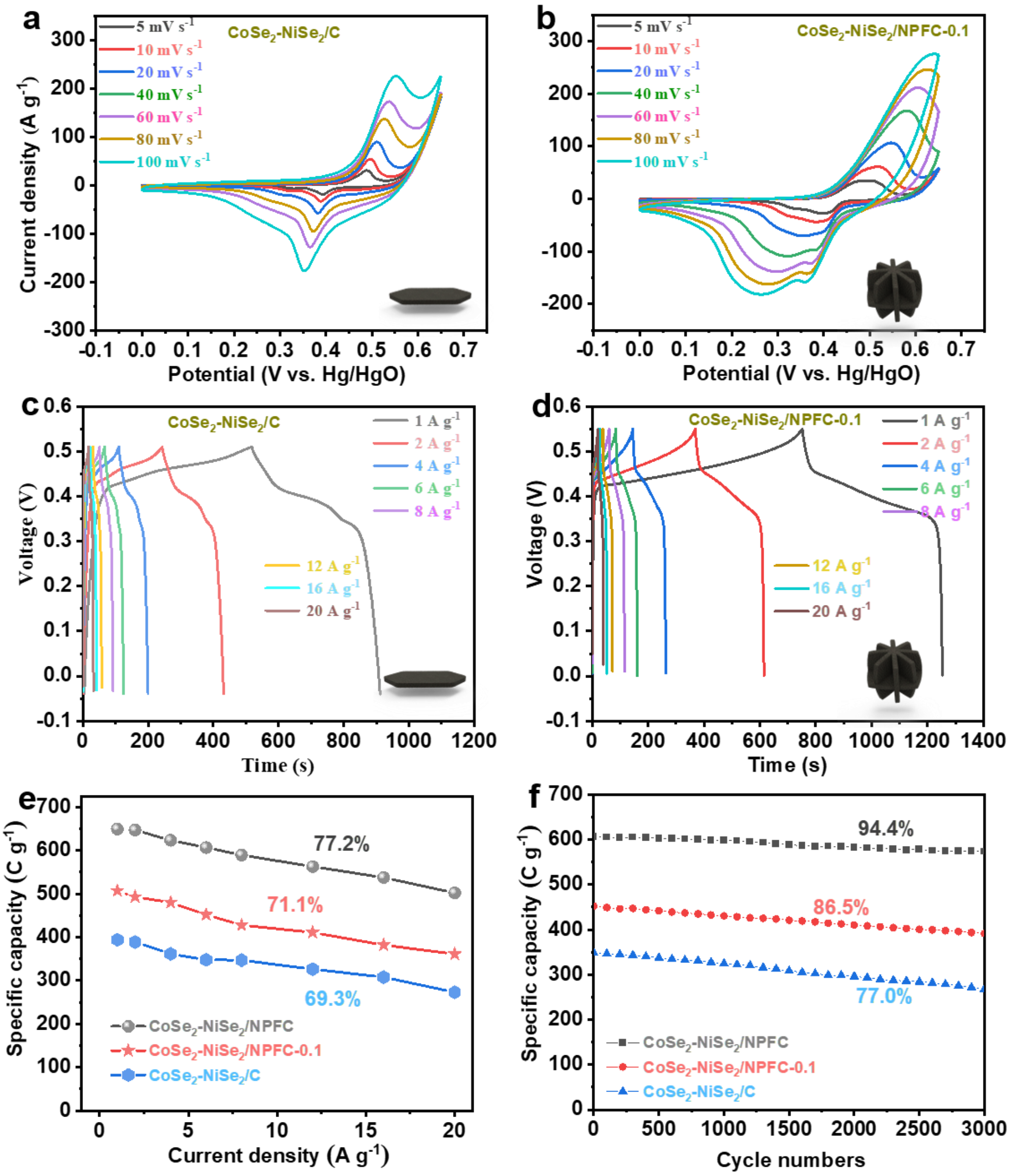

Figure S14. The CV of (a) $CoSe_2$-$NiSe_2$/C and (b) $CoSe_2$-$NiSe_2$/NPFC-0.1; The GCD of (c) $CoSe_2$-$NiSe_2$/C and (d) $CoSe_2$-$NiSe_2$/NPFC-0.1; (e)The Rate performance of $CoSe_2$-$NiSe_2$/NPFC, $CoSe_2$-$NiSe_2$/NPFC-0.1 and $CoSe_2$-$NiSe_2$/C; (f) the durability performance of $CoSe_2$-$NiSe_2$/NPFC, $CoSe_2$-$NiSe_2$/NPFC-0.1 and $CoSe_2$-$NiSe_2$/C.

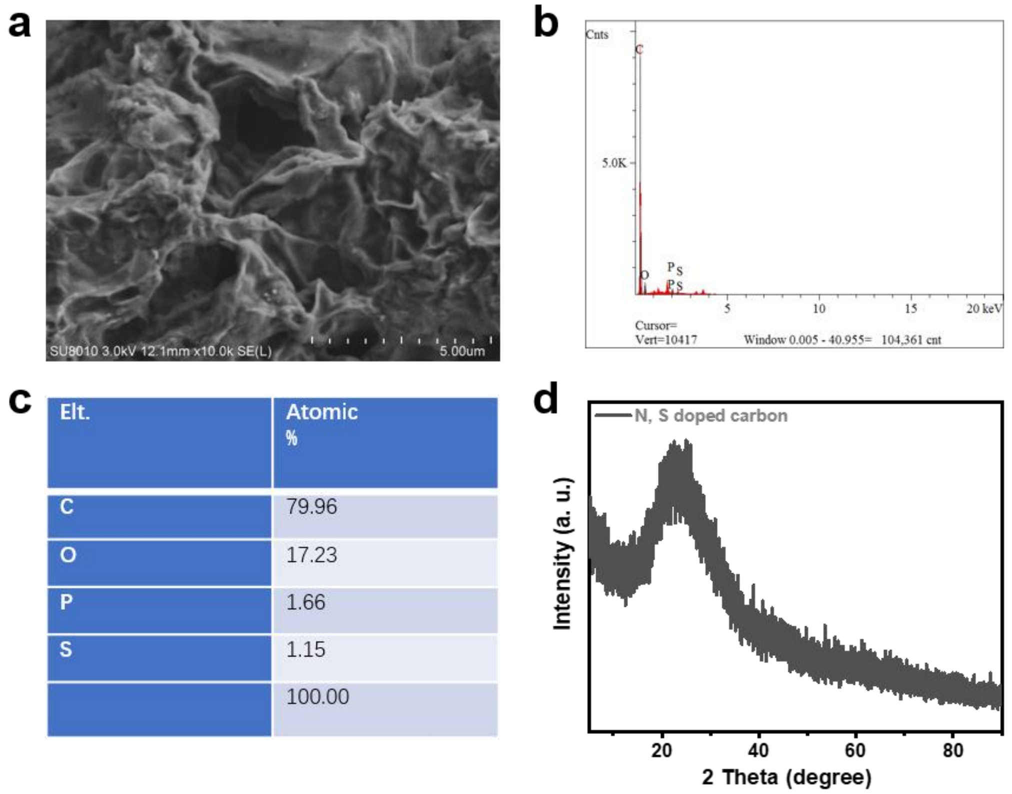

Elt.	Atomic %
C	79.96
O	17.23
P	1.66
S	1.15
	100.00

Figure S15. The (a) SEM, (b) EDS, (c) Elemental ratios and (d) XRD of N, S-doped carbon.

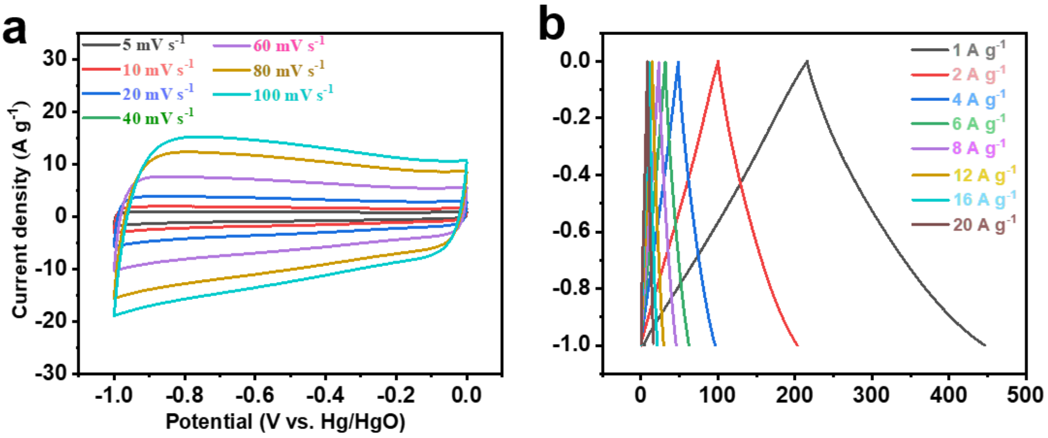

Figure S16. The (a) CV and (b) GCD of Active carbon.

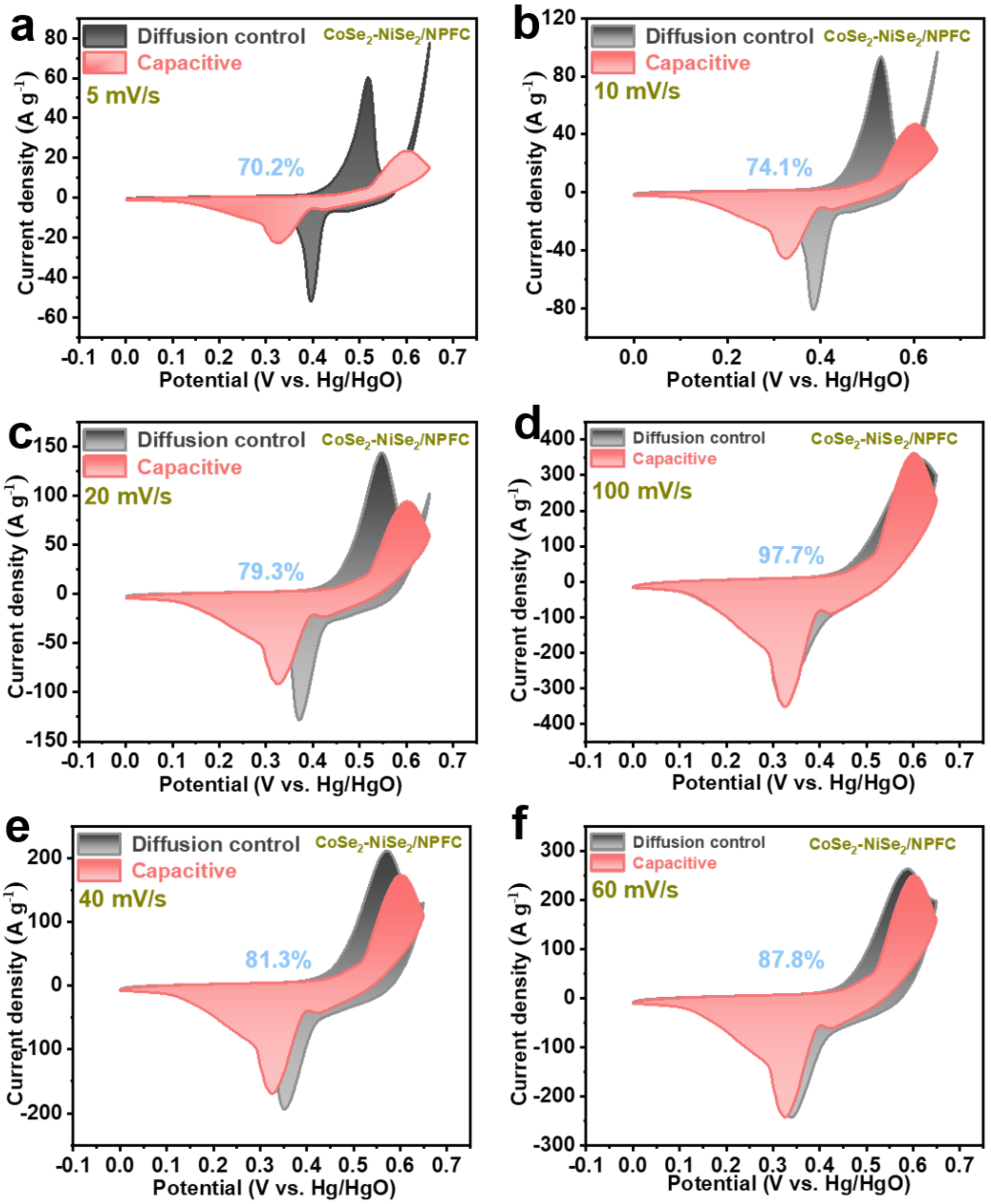

Figure S17. (a-f) The contribution of the capacitance of $CoSe_2$-$NiSe_2$/NPFC to the total current at different scan rates.

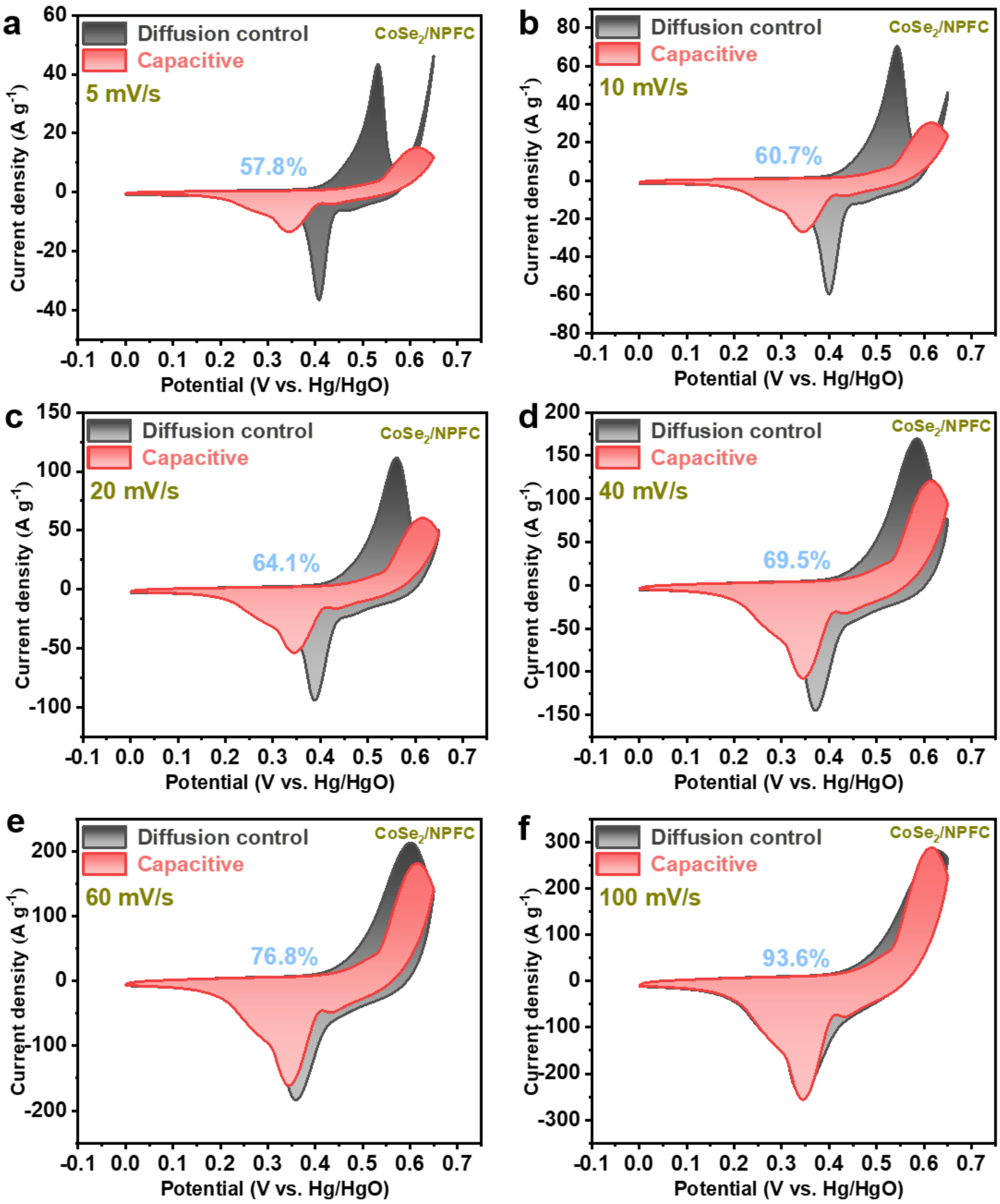

Figure S18. (a-f) The contribution of the capacitance of $CoSe_2$/NPFC to the total current at different scan rates.

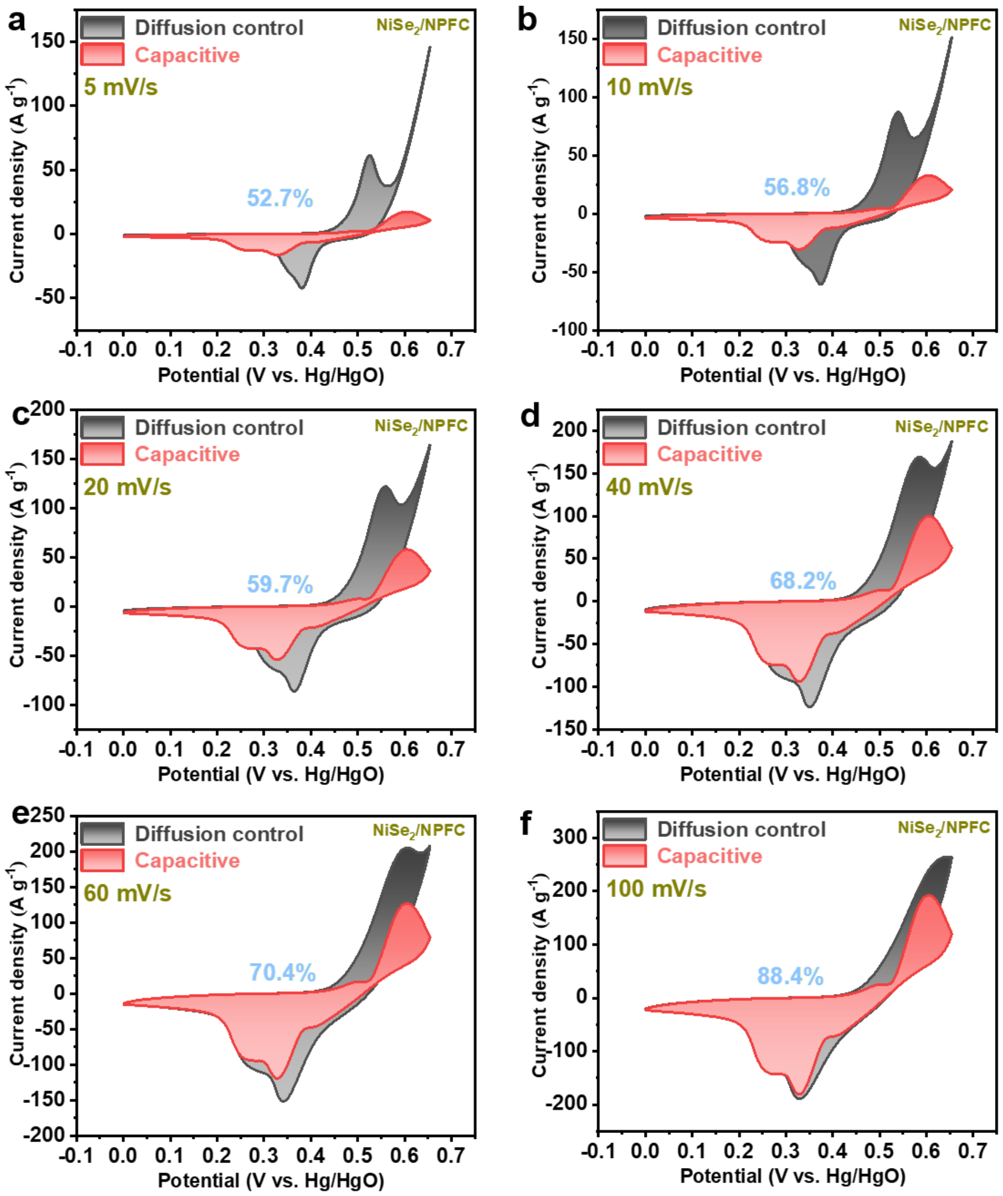

Figure S19. (a-f) The contribution of the capacitance of $NiSe_2$/NPFC to the total current at different scan rates.

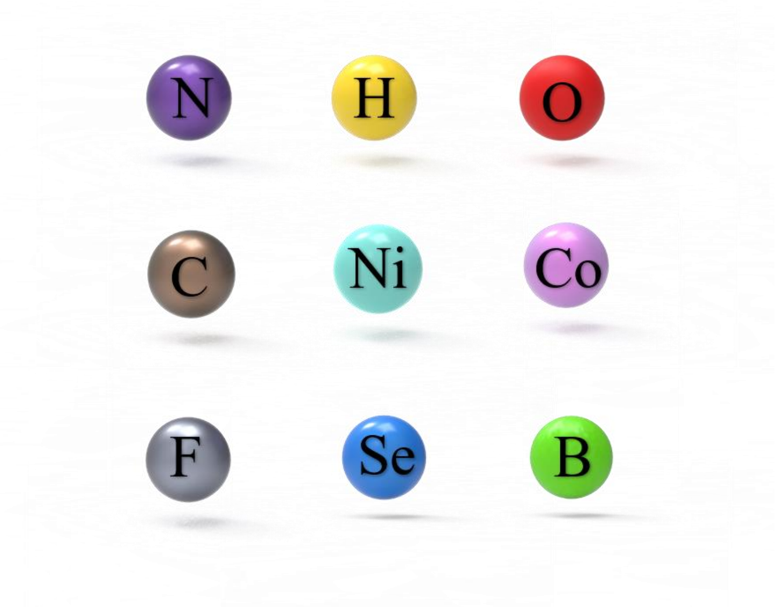

Figure S20. Explanation of each ball.

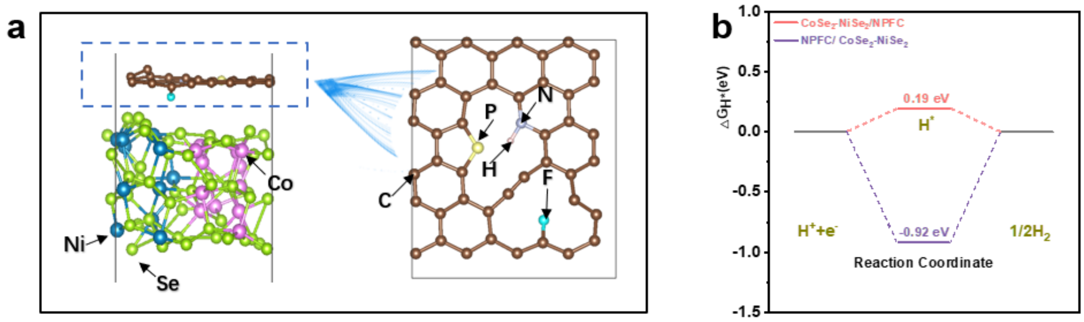

Figure S21. The model of (a) NPFC/$CoSe_2$-$NiSe_2$ and (b) the ΔG_{H*} of NPFC/$CoSe_2$-$NiSe_2$ and $CoSe_2$-$NiSe_2$/NPFC.

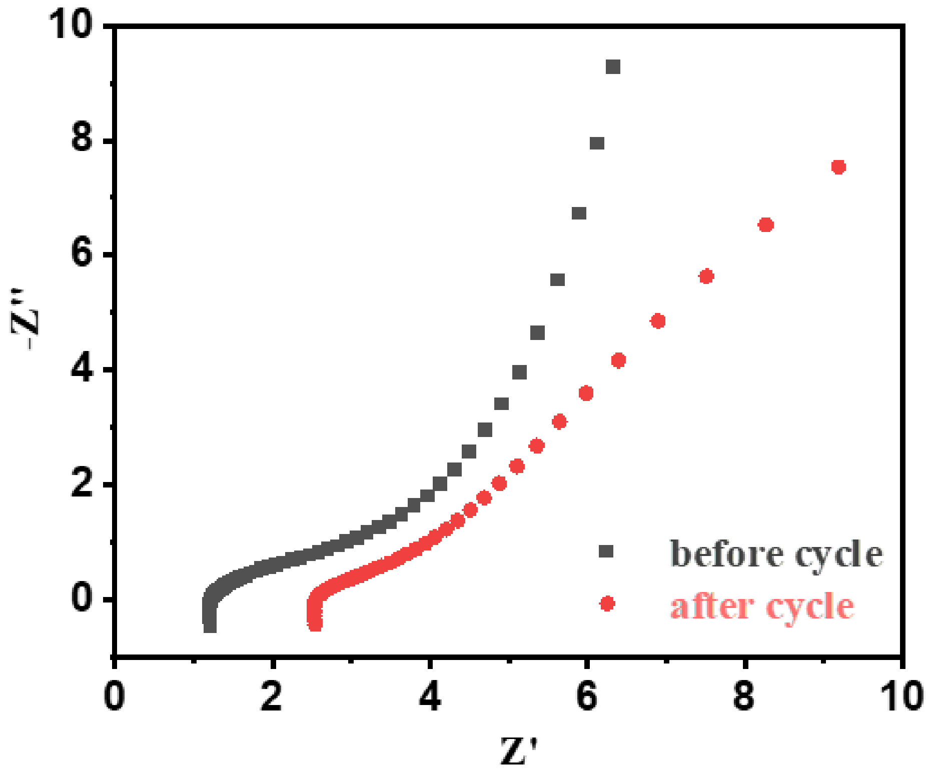

Figure S22. CP of $CoSe_2$-$NiSe_2$/NPFC//S, P-doped carbon before and after cycling.

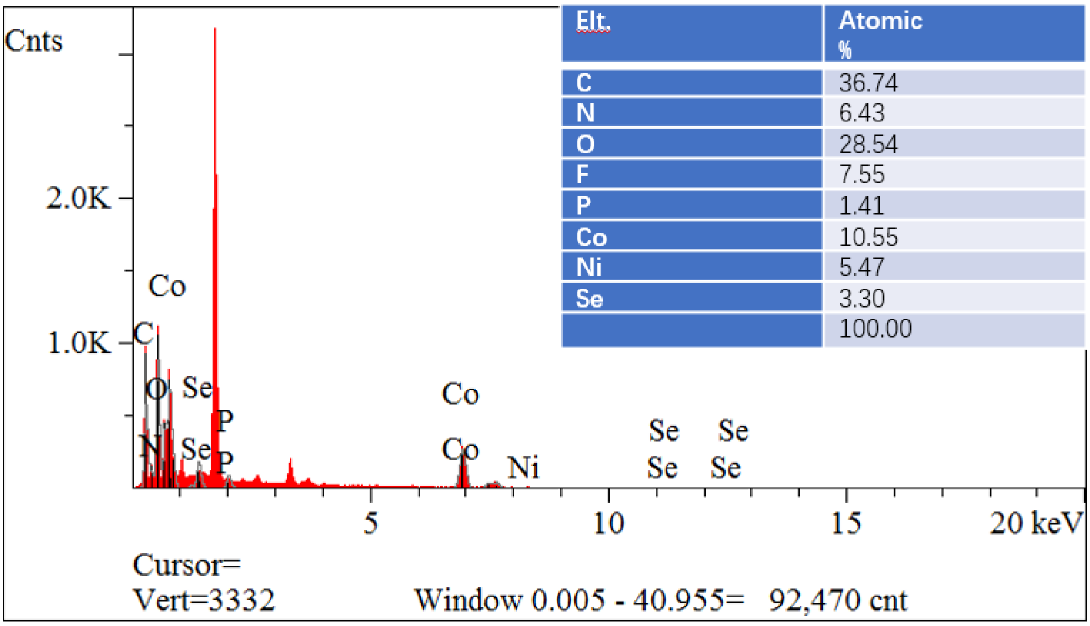

Elt.	Atomic %
C	36.74
N	6.43
O	28.54
F	7.55
P	1.41
Co	10.55
Ni	5.47
Se	3.30
	100.00

Figure S23. The EDS after cycling.

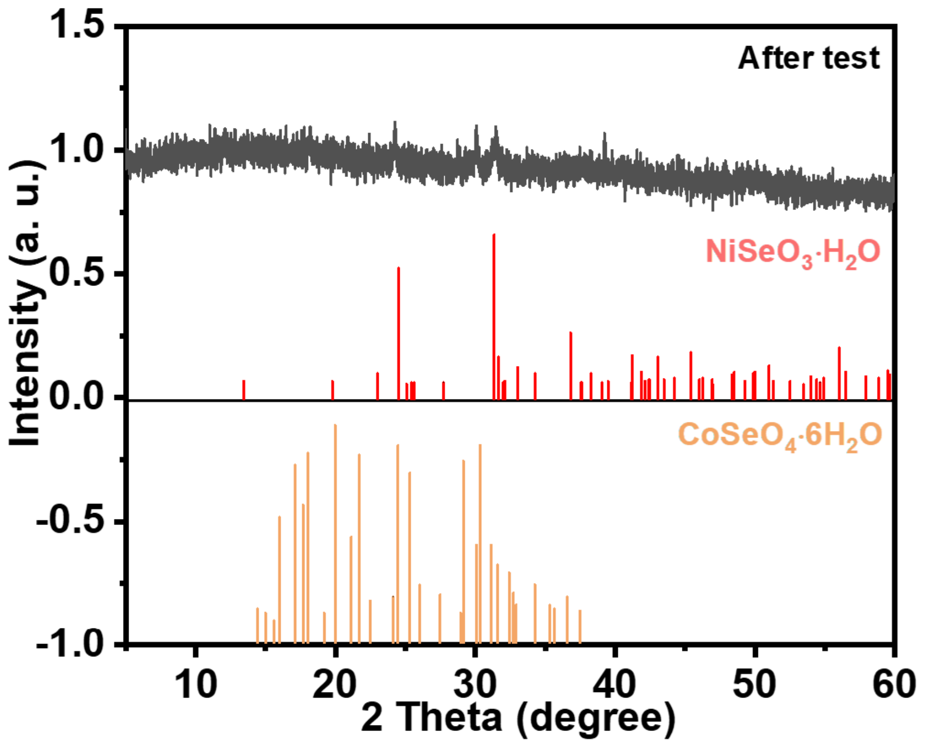

Figure S24. The XRD after cycling.

Table S1. EXAFS fitting parameters at the M K-edge for various samples.

Sample	Shell	N^a	$R(\text{Å})^b$	$\sigma^2\times10^3(\text{Å}^2)^c$	ΔE_0 (eV)^d	R factor
Ni K-edge (S_0^2=0.703)						
Ni foil	Ni-Ni	12*	2.483±0.003	6.2±0.3	7.0±0.6	0.0024
$NiSe_2$/NPFC -Ni	Ni-Se	6.7±0.1	2.478±0.001	5.8±0.2	1.0±0.4	0.0009
$CoSe_2$-$NiSe_2$/NPFC -Ni	Ni-Se	6.3±0.2	2.458±0.004	7.5±0.5	0.7±1.0	0.0039
Co K-edge (S_0^2=0.783)						
Co foil	Co-Co	12*	2.490±0.002	6.2±0.3	7.5±0.4	0.0015
$CoSe_2$ /NPFC -Co	Co-Se	6.0±0.2	2.180±0.051	6.9±1.3	2.5±1.2	0.0007
$CoSe_2$-$NiSe_2$/NPFC -Co	Co-Se	5.7±0.1	2.304±0.008	7.6±1.0	1.6±1.7	0.0096

$^a N$: coordination numbers; $^b R$: bond distance; $^c\sigma^2$: Debye-Waller factors; ^{d} ΔE_0: the inner potential correction. R factor: goodness of fit.

Table S2. Electrocatalytic activity comparison of $CoSe_2$-$NiSe_2$/NPFC for HER in 0.5 M H_2SO_4 solution with reported HER catalysts.

**Catalyst**	**η(mV) at**	**Tafel**	**Ref.**

	**j = 10 mA/cm^2**	**slope (mV/dec)**	
**$CoSe_2$-$NiSe_2$/NPFC**	**57**	**35**	**This work**
$NiCo_2Se_4$-NWs/NCNFs	200	55.9	*Electrochim. Acta, 2020, 333, 135515*
$Mn_{0.05}$Co0$_{.95}$$Se_2$	195	36	*J. Am. Chem. Soc., 2016, 138, 5087−5092*
$Co_{0.9}Ni_{0.1}Se$	185.7	58	*Angew. Chem. Int. Ed.* **2020**, *59*, 22743–22748
CoSe@NCNT/NCN	197	43	*Electrochim. Acta* **2020**, *337*, 135685
$CoSe_2$/Ti	110	48.5	*J. Mater. Chem. A* **2016**, *4*, 4553−4561
$CoSe_2$/HOPG	272	61	*ACS Energy Lett.* **2016**, *1*, 607−611
$CoSe_2$@carbon nanotubes	132	82	*Nano Energy* **2016**, *28*, 143−150
$CoSe_2$@graphene/GC	210	42	*Chem. Eng. J.* **2017**, *321*, 105−112
$CoSe_2$/GC	150	51	*Nanotechnology* **2017**, *28*, 315401
$CoSe_2$-carbon fiber/GC	133	-	*J. Alloy Compd.* **2017**, *702*, 611−618
$CoSe_2$ NW/carbon cloth	130	32	*ACS Appl. Mater. Interfaces* **2015**, *7*, 3877−3881

Table S3. Electrocatalytic activity comparison of Catalyst $CoSe_2$-$NiSe_2$/NPFC for HER in 1.0 M KOH solution with reported HER catalysts.

Catalyst	ŋ(mV) at j=10 mA/cm^2	Tafel slope (mV/dec)	Ref.
**$CoSe_2$-$NiSe_2$/NPFC**	**86**	**28**	**This work**
$MoSe_2$@$NiCo_2Se_4$	89	85	*Small **2022**, 18, 2200622*
$Co_{0.75}Ni_{0.25}Se$/NF	106	74	*Nanoscale **2019**, 11, 7959.*
$NiSe_2$@NC	162	10	*ACS Appl. Mater. Interfaces* **2020**, *12*, 28288−28297
F-CoS_2@NF	112	61	*Small* **2019**, *15*, 1904670
c-$CoSe_2$/CC	190	85	*Adv. Mater.* **2016**, *28*, 7527−7532
Ni/NiS	230	115	*Adv. Funct. Mater.* **2016**, *26*, 3314−3323
MoS_2/$MoSe_2$	235	96	*Nano Energy* **2018**, *48*, 337−344
Co_9S_8@MoS_2	177	84	*J. Catal.* **2020**, *385*, 129−139
$MoSe_2$/SnS_2	285	109	*Nat. Commun.* **2019**, *10*, 982
Co-MoS_2/Mo_2CT_x	112	82	*Appl. Catal. B* **2019**, *254*, 432−442
Co_2P/Co-Foil	154	59	*J. Mater. Chem. A* **2017**, *5*, 10561−10566
CoP@NPMG	151	75	*Nanoscale* **2018**, *10*, 2603−2612

Table S4. Electrochemical performance comparison of $CoSe_2$-$NiSe_2$/NPFC for supercapacitors with other reported materials.

**Materials**	**Energy density and power density**	**Ref.**
**$CoSe_2$-$NiSe_2$/NPFC**	**40.8 Wh kg^{-1} at 750 W kg^{-1}**	**This work**
$Ni_{0.85}Se$@$MoSe_2$	19.3 Wh kg^{-1} at 3900 W kg^{-1}	*J. Mater. Sci.* **2018**, *53*, 3647–3660
$(Ni_xCo_{1-x})_9Se_8$	17 Wh kg^{-1} at 3.1 kW kg^{-1}	*Adv. Energy Mater.* **2018**, *8*, 1801392
GNR/$Co_{0.85}Se$	18.5 Wh kg^{-1} at 6.77 kW kg^{-1}	*Adv. Funct. Mater. 29 (2019) 1904182*
Ni_3Se_2 NSs@CF	32.8 Wh kg^{-1} at 677.03 W kg^{-1}	*Adv. Energy Mater.* **2017**, *7*, 1601362
$NiCo_2(S_xSe_{1-x})_5$	47.2 Wh kg^{-1} at 801 W kg^{-1}	*Chem. Eng. J.* **2018**, *351*, 678–687
$(Ni_{0.33}Co_{0.67})Se_2$ CHSs	21.1 Wh kg^{-1} at 400 W kg^{-1}<br>29.1 Wh kg^{-1} at 800 W kg^{-1}	*Electrochim. Acta* **2018**, *281*, 109-116
NiCoP/NPC	40.2 Wh kg^{-1} at 800 W kg^{-1}<br>26.8 Wh kg^{-1} at 7973 W kg^{-1}	*Appl. Catal. B* **2021**, *283*, 119635
NiCoP nanoplates	32.9 Wh kg^{-1} at 1301 W kg^{-1}	*Nano Energy* **2017**, *35*, 331–340
Ni_3Se_2 NSs@CF	22.1 Wh kg^{-1} at 6.0 kW kg^{-1}	Adv. Energy Mater. 7 (2017) 1601362.

References

[1] Kresse, G.; Furthmüller, J. Efficiency of Ab-Initio Total Energy Calculations for Metals and Semiconductors Using a Plane-Wave Basis Set. Comput. Mater. Sci. 199, 6, 15−50.
[2] Kresse, G.; Furthmüller, J. Efficient Iterative Schemes for Ab Initio Total-Energy Calculations Using a Plane-Wave Basis Set. Phys. Rev. B 1996, 54, 11169−11186.

[3] Perdew, J. P.; Burke, K.; Ernzerhof, M. Generalized Gradient Approximation Made Simple. Phys. Rev. Lett. 1996, 77, 3865−3868.
[4] Kresse, G.; Joubert, D. From Ultrasoft Pseudopotentials to the Projector Augmented-Wave Method. Phys. Rev. B 1999, 59, 1758-1775.
[5] Blöchl, P. E. Projector Augmented-Wave Method. Phys. Rev. B 1994, 50, 17953−17979.
[6].Grimme, S.; Antony, J.; Ehrlich, S.; Krieg, H. J. Chem. Phys. 2010, 132, 154104.
[7].H. J. Monkhorst and J. D. Pack, Phys. Rev. B, 1976, 13, 5188-5192.
[8] J. Rossmeisl, A. Logadottir, J. K. Nørskov, Chem. Phys. 319 (2005) 178-184.
[9] J. K. Nørskov, J. Rossmeisl, A. Logadottir, L. R. K. J. Lindqvist, J. R. Kitchin, T. Bligaard, H. Jonsson, J. Phys. Chem. B 108 (2004) 17886-17892.
